# Age and Date for Early Arrival of the Acheulian in Europe (Barranc de la Boella, la Canonja, Spain)

**DOI:** 10.1371/journal.pone.0103634

**Published:** 2014-07-30

**Authors:** Josep Vallverdú, Palmira Saladié, Antonio Rosas, Rosa Huguet, Isabel Cáceres, Marina Mosquera, Antonio Garcia-Tabernero, Almudena Estalrrich, Iván Lozano-Fernández, Antonio Pineda-Alcalá, Ángel Carrancho, Juan José Villalaín, Didier Bourlès, Régis Braucher, Anne Lebatard, Jaume Vilalta, Montserrat Esteban-Nadal, Maria Lluc Bennàsar, Marcus Bastir, Lucía López-Polín, Andreu Ollé, Josep Maria Vergés, Sergio Ros-Montoya, Bienvenido Martínez-Navarro, Ana García, Jordi Martinell, Isabel Expósito, Francesc Burjachs, Jordi Agustí, Eudald Carbonell

**Affiliations:** 1 Institut Català de Paleoecologia Humana i Evolució Social (IPHES), Tarragona, Spain; 2 Àrea de Prehistòria, Departament d’Història i Història de l’Art, Facultat de Lletres. Universitat Rovira i Virgili (URV), Tarragona, Spain; 3 Grupo Quaternário e Pré-História do Centro de Geociências (GQP-CG), Faculdade de Ciências e Tecnologia, Universidade de Coimbra (UC), Coimbra, Portugal; 4 Departamento de Paleobiología, Museo Nacional de Ciencias Naturales (MNCN), Consejo Superior de Investigaciones Científicas (CSIC), Madrid, Spain; 5 Laboratorio de Paleomagnetismo. Departamento de Física, Escuela Politécnica Superior, Universidad de Burgos (UBU), Burgos, Spain; 6 Área de Prehistoria, Departamento de Ciencias Históricas y Geografía, Universidad de Burgos (UBU), Burgos, Spain; 7 Laboratoire National des Nucléides Cosmogéniques, Centre de Recherche et d’Enseignement de Géosciences de l’Environnement (CEREGE), Université Aix-Marseille (UAM), Centre National de la Recherche Scientifique (CNRS-UM34), Aix-en-Provence, France; 8 Museo de Prehistoria y Paleontología, Orce, Granada, Spain; 9 Institució Catalana de Recerca i Estudis Avançats (ICREA), Barcelona, Spain; 10 Departament d’Estratigrafia, Paleontologia i Geociències Marines, Facultat de Geologia, Universitat de Barcelona (UB), Barcelona, Spain; 11 Visiting professor, Institute of Vertebrate Paleontology and Paleoanthropology of Beijing (IVPP), Beijing, China; 12 Unit associated to Consejo Superior de Investigaciones Científicas (CSIC), Departamento de Paleobiología. Museo Nacional de Ciencias Naturales (MNCN), Madrid, Spain; University of Oxford, United Kingdom

## Abstract

The first arrivals of hominin populations into Eurasia during the Early Pleistocene are currently considered to have occurred as short and poorly dated biological dispersions. Questions as to the tempo and mode of these early prehistoric settlements have given rise to debates concerning the taxonomic significance of the lithic assemblages, as trace fossils, and the geographical distribution of the technological traditions found in the Lower Palaeolithic record. Here, we report on the Barranc de la Boella site which has yielded a lithic assemblage dating to ∼1 million years ago that includes large cutting tools (LCT). We argue that distinct technological traditions coexisted in the Iberian archaeological repertoires of the late Early Pleistocene age in a similar way to the earliest sub-Saharan African artefact assemblages. These differences between stone tool assemblages may be attributed to the different chronologies of hominin dispersal events. The archaeological record of Barranc de la Boella completes the geographical distribution of LCT assemblages across southern Eurasia during the EMPT (Early-Middle Pleistocene Transition, circa 942 to 641 kyr). Up to now, chronology of the earliest European LCT assemblages is based on the abundant Palaeolithic record found in terrace river sequences which have been dated to the end of the EMPT and later. However, the findings at Barranc de la Boella suggest that early LCT lithic assemblages appeared in the SW of Europe during earlier hominin dispersal episodes before the definitive colonization of temperate Eurasia took place.

## Introduction

**Figure 1:**
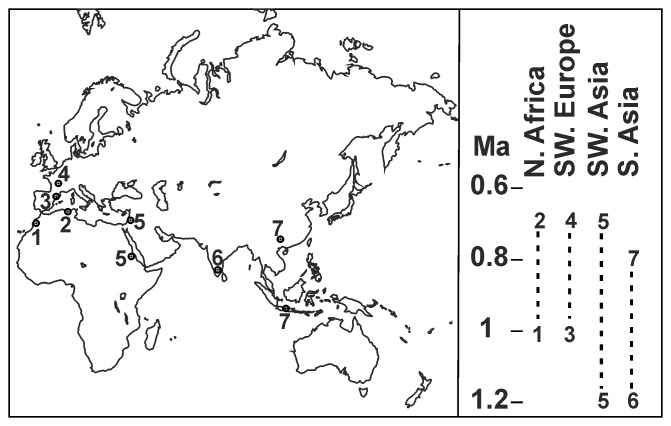
Geographical and temporal distribution of key Acheulian sites out of sub-Saharan Africa. Keys: 1, Thomas Quarry 1 - Casablanca, Morocco; 2, Ternifine and Errayah, Algeria; 3, Barranc de la Boella, Spain; 4, La Noira, France; 5, Ubeidiya and Gesher Benot Yaaqov, Israel; and Buia, Eritrea; 6, Attirampakkam and Isampur, India; 7, Sangiran, Java; and Bose, China.

Among the results of the research devoted to the cultural-stratigraphic entities of the Lower Palaeolithic, one of the most noteworthy and debatable concerns is the temporal and geographic distribution of large cutting tools (LCT) as trace fossils of the Acheulian [1]–[8]. Prehistoric lithic assemblages containing sets of LCT and reduction sequences intended to produce large flakes is a broad taxonomic description of the Acheulian [3], [9]–[11]. However, in Africa the presence of LCT is not strictly related to Acheulian industry, since this type of tool already appeared in the Developed Oldowan tradition, although authors such as [12], [13], support the suggestion made by others previously that the Developed Oldowan is actually Acheulian. For Eurasia, the Pebble and Core (PBC) technological assemblages [14], found in western Asia around 2 Ma [15] are consistently considered to be the earliest known lithic artefacts, while in Europe the earliest sites are no older than 1.2 or 1.5 Ma [16]. Given that Acheulian industry is present in Asia at around 1.4 and 1.2 Ma in both the Levantine Corridor [17] and India [18], [19] (Figure 1), researchers have had to reconsider the existence of LCT as trace fossils in the taxonomic identity of the Acheulian [3]. This empirical concern about the European Lower Palaeolithic record suggests that PBC assemblages (Clactonian, Tayacian, etc.) have the same taxonomic identity as the LCT assemblages and are considered to reflect a variation in behaviour or land use, as has been described in many studies of the Early Pleistocene archaeological record at Awash basin, Olduvai and Ubeidiya, and as Leakey proposed for the Developed Oldowan [7], [20]–[22]. Conversely, specific stone-craft traditions have been recognised in the stratigraphic interdigitation of Clactonian and Acheulian assemblages in Britain [23]. There appears to be a pattern in the distribution of Lower Palaeolithic artefact assemblages in Europe [24]: Acheulian industry is present in the south and west and absent in the north and east. The uniqueness of Acheulian industry is not explained merely by the equifinality of the material culture of different human groups and their land use strategies [9]. In the long occupational sequence at Gesher Benot Ya’aqov, bifaces seem to reflect a technical tradition, due to their unusual homogeneity compared to other types of tools. The presence of cleavers made from large flakes throughout the circum-Mediterranean area also seems to point to a Middle Pleistocene Acheulian technological tradition [10], [11]. Picks and “trifacial” knapping have also been considered to be within the Middle Pleistocene Acheulian morphotypes and are found in North Africa, on the Iberian Peninsula and in south-eastern France [2], [25]. The temporal distribution of this southern Acheulian (meridional Acheulian) industry is probably the same as that of the central European Acheulian, and the differences between them are considered to be one of the fundamental arguments suggesting that there was a Middle Pleistocene prehistoric corridor over the Strait of Gibraltar [6], [10], [11], [26].

The Barranc de la Boella lithic assemblage record contains few LCT morphotypes. Ratios of LCT are considered to be a criterion for identifying Acheulian variants in Africa [2], [3]. However, the mere presence of LCT in the European record has been considered sufficient to characterize the industry as Acheulian [8].The Barranc de la Boella evidence reported in this study focuses on stratigraphy with the aim of defining the geochronologic and chronostratigraphic units in the sedimentary deposits containing the LCT assemblage. We attempt to review and discuss our results in terms of the chronology of the Palaeolithic record in the Iberian Peninsula and the areas around the Mediterranean Sea dating to 942–641 ka (Marine Isotopic Stages 25 to 16) or the Early-Middle Pleistocene transition (EMPT) [24], [27]–[29]. The chronostratigraphic horizon and age of the artefact assemblage of the Barranc de la Boella record confirms the much debated presence of LCTs in the Palaeolithic record of the Early Pleistocene in Europe [30]–[32]. However, our concern is to avoid excessive emphasis on taxonomic cultural preconceptions which are based on lithic assemblages, and emphasise, instead, chronology as important primary data in archaeological research. Consistent stratigraphic studies may resolve conceptual problems with the taxonomy and nomenclature of the Lower Palaeolithic record. Finally, we point out the chronological inconsistencies between the fluvial terrace archives and the Lower Palaeolithic records of the Iberian Peninsula and the chronology of the archaeological findings from Barranc de la Boella. A detailed description of the Barranc de la Boella artefact assemblage will be given separately in a future, specifically technological, study.

## Materials

The Barranc de la Boella site (la Canonja, Spain) is located in the north-east of the Iberian Peninsula, 3 km from the Mediterranean coast near the city of Tarragona, and is part of the terrace system of the lower Francolí River basin. The Boella area is located at the foot of the Neogene monocline relief of the Gavarres massif at 55 m above the level of the Mediterranean Sea. The lower Francolí River basin constitutes a half-graben, sub-parallel to the north-eastern coast of the Iberian Peninsula and sunken from the common base level of the basins of the Catalan Coastal Ranges [33].

**Figure 2:**
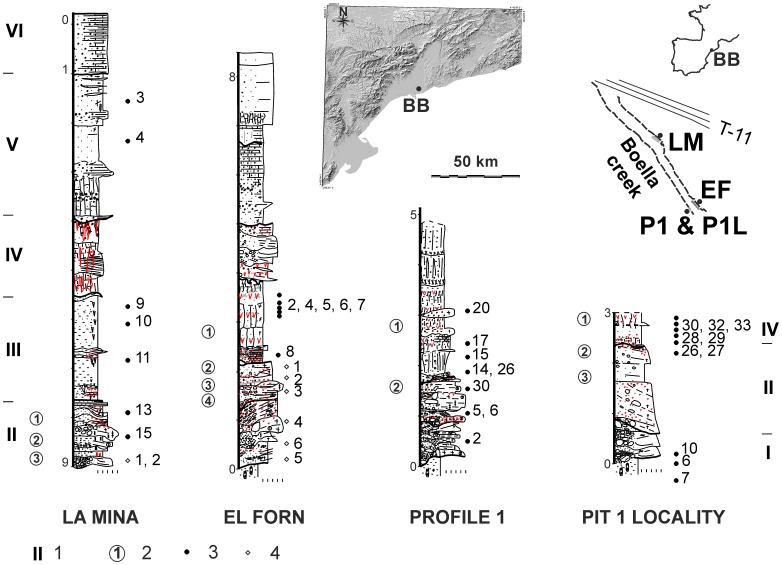
Lithostratigraphic units, archaeopalaeontological levels and position of sedimentary samples for palaeomagnetic and cosmogenic studies at Barranc de la Boella localities. Keys: 1, lithostratigraphic unit; 2, archaeological layer; 3, stratigraphic position and number of reliable palaeomagnetic samples mesured; 4, stratigraphic position and number of cosmogenic dating samples (see Table 3).

Since 2007, fieldwork at Barranc de la Boella has been conducted since 2007 in four main locations (Figure 2): Pit 1 Locality (P1L); Profile 1 (P1); Pit 2 or la Mina (LM); Pit 3 or el Forn (EF). P1L was excavated in 2007 following the discovery of proboscidean dental remains. These remains are a continuation of the skeletal elements found and published by Dr. S. Vilaseca [34]. P1L consists of 10 m2 of excavated area. P1 is an outcrop near P1L chosen to characterize the stratigraphy of the upper beds affected by recent anthropogenic disturbance. LM is located 180 m upstream (Figure 2). EF is located in front of P1L on the opposite bank of the creek. LM and EF have been analysed within an excavated area of 25 m2 each.

The 9 m thick sedimentary succession at Barranc de la Boella contains six lithostratigraphic units (Figure 2). Unit I overlays unconformably a stratum of Neogene clays, and consists of a 1.5 m bed-set of stratified clast-supported coarse gravel. Unit II is 2 m thick and contains poorly stratified sand and gravel. The lithology of unit III is red-mottled massive mud with a thickness of 2 m. Units IV and V contain massive mud ranging from 2 to 5 m thick. These units show impregnative and rhizospheric accumulations of calcium carbonate. Unit VI is made up of horizontal beds of gravel and red-yellow sandy clay with a total thickness of 2 m. Unit VI contains a petrocalcic horizon that continues across the alluvial fan surfaces of the Middle and Upper Pleistocene [35]. From a geomorphological point of view, stratigraphic units I to V are within an incised valley encased in the fluvial terrace at +60 m and stratified with the terrace at +50 m in the lower valley of the Francolí River basin.

The palaeomagnetic and rock magnetic study of the sediments was conducted on oriented cores collected from the four main localities in lithostratigraphic units I to VI (Figure 2). Cosmogenic nuclide samples were taken from units I and II in the EF locality and unit II in the LM locality (Figure 2).

Remains of biological origin (including large and small mammals, malacofauna and plant pseudomorphs), and lithic industries are distributed mainly in stratigraphic units II and III. The main mammals taxa identified in the Barranc de la Boella localities are *Mammuthus meridionalis*, *Hippopotamus antiquus* and *Dama* cf. *vallonetensis* (Table 1). The list of fauna includes the small mammals *Mimomys savini* and *Victoriamys chalinei*. A large bone-eating carnivore has been detected through coprolites, which may correspond to the large hyena cf. *Pachycrocuta brevirostris*. The biostratigraphy of small mammals indicates a temporal span set between the late Early Pleistocene and the early Middle Pleistocene [36] (Information S1 and S2).

**Table 1:** The taxonomic faunal list and presence in the Baranc de la Boella localities (P1L; pit 1 locality; EF, el Forn; and LM, la Mina), lithostratigraphic units and archaeopaleontological levels.

BB Localities	P1L		EF							LM		
Lithostratigraphic unit	II		III	II						II		
Archaeopalaeontological level	2	3	1	2	3	4	5	6	7	1	2	3
*Cervus* sp.			x	x	x	x		x			x	
*Dama* cf. *vallonetensis*	x	x	x	x	x	x	x			x	x	x
*Megaloceros savini*			x	x	x	x					x	
*Equus* cf. *stenonis*		x	x	x	x	x	x			x	x	
Bovini indet.				x	x	x				x		
*Hippopotamus antiquus*			x	x	x	x	x			x	x	x
*Stephanorhinus hundsheimiensis*			x	x	x	x			x			
*Mammuthus meridionalis*	x	x		x	x	x					x	
*Sus strozzi*			x									
*cf. Pachycrocuta brevirostris*					x							
*Ursus* sp.						x				x	x	
*Canis* sp.											x	
cf *Panthera gombaszoegensis*											x	
*Macaca silvana*											x	
*Castor* sp.						x						
*Mimomys savini*	x			x	x						x	
*Victoriamys chalinei*											x	

The Barranc de la Boella level 2 at the P1L, EF and LM localities contains largest number of lithic remains (Table 2). Level 2 at P1L and EF is set on top of a bed 0.2–0.3 m thick and composed of matrix supported coarse gravels by muddy sand and located in the upper part of lithostratigraphic unit II (Figure 2). The LM locality has 3 levels of faunal and lithic remains within micro-stratified beds of massive to graded pebbly sand and sandy mud-supported gravel thinning upwards (Figure 2). The levels 5, 6 and 7 at EF and level 3 at P1L are only palaeontological and exhumed within clast-supported coarse gravel bed sets (Table 1). Unit III has only one level (level 1) at the EF and P1L localities. The archaeopalaeontological levels depicted in the field have been verified by plotting spatial coordinates (x, y, z) of the faunal and lithic remains using the *ARCHePlotter* software.

**Table 2:** Lithic industry categories per Barranc de la Boella localities (P1L; pit 1 locality; EF, el Forn; and LM, la Mina), lithostratigraphic units and archaeopaleontological levels.

BB localities	P1L			EF				LM		
Lithostratigraphic unit	III	II		III	II			II		
Archaeological level	1	2	3	1	2	3	4	1	2	3
Hammerstones		3		2	2	2	1			2
Pebbles fractured	2	7	2	3	5	5	5	1	7	
LCT		1			1		1?			
Chopping tools					1				1	
Choppers						1	1		3	
Cores		3	1	2	5	2	1		4	1
Small tools		8	2		4	2	2	2	5	1
Flakes	11	45	1	3	30	4	11	5	16	2
Broken flakes & flake fragments	3	37		1	9	3	3	2	13	5
Fragments	1	20	2	1	9	3	9		5	1
Indeterminated		1								
**Total general**	17	125	8	12	66	22	34	10	54	12

The sedimentary facies associations and palaeontological record documented in units II and III are related to flooded habitats. The unit II lithofacies, with its poorly stratified sands and gravels, indicates subaerial and subaquatic mass flow deposits which are often described in alluvial fan and fan-delta sedimentary environments [37]. An aquatic environment is further suggested by the presence of hippopotamuses and water voles found in the LM and EF localities. Current geological risk cartography in the Francolí River valley depicts similar flooded zones [38]. As in the eastern sector of the Francolí River valley, currently flooded areas are located close to tributary confluences with the Francolí River. The areas affected by modern’s day torrential flooding dynamics have little aerial extension within the Pleistocene cartography of the lower Francolí River basin. The Early Pleistocene occupation of analogous flooded areas suggests a hominin preference for this habitat type as is also suggested by other studies of the palaeoecology of Lower Palaeolithic hominins [39].

## Methods

The samples of sediments collected for magnetic and cosmogenic studies were collected from the four main localities during the 2007 to 2012 fieldwork authorized by Servei d’Arqueologia i Paleontologia of the Generalitat de Catalunya (Spain) (Figure 2). Field studies at Barranc de la Boella archaeological project did not involve endangered or protected species. The UTM coordinates at the main pit area explored, or Pit 1 locality, are:

UTM31N – ETRS89 346467.9 E 4555318.1 N

UTM31N – ED50 346519.9 E 4555454.9 N

GEOGRAPHIC – ETRS89 1.170269 41.134047.

The palaeomagnetic study comprised the analysis of oriented cores extracted using an electric drill and oriented in the field using a magnetic compass. The analyses were carried out at the Palaeomagnetism Laboratory of the University of Burgos (Spain). Natural remanent magnetization (NRM) was measured using a 2G superconducting cryogenic magnetometer (noise level ∼ 5 x 10^−12^ Am^2^). NRM stability was analysed by both progressive stepwise thermal demagnetization and alternating field (AF) demagnetization. Progressive stepwise thermal demagnetization was carried out up to 686°C using a TD48-SC (ASC) thermal demagnetizer. AF demagnetization was performed stepwise up to a maximum peak field of 100 milliTesla (mT) using a 2G-600 automatic sample degaussing system. In order to assess possible mineralogical changes, magnetic susceptibility was measured at room temperature after each step in the thermal demagnetization. Additionally, in order to characterize the magnetic mineralogy and magnetic properties in more detail, rock-magnetic experiments were conducted on pilot samples from each stratigraphic unit with the aid of a variable field translation balance (MMVFTB, UBU laboratory). These consisted of: i) isothermal remanent magnetization (IRM) progressive acquisition curves; ii) hysteresis loops (±1 T); iii) back-field curves; and iv) Curie curves up to 700°C in air.

The Barranc de la Boella sediments have been analysed in order to obtain radiometric dates by using the radioactive decay of the two cosmogenic nuclides (^26^Al and ^10^Be) that are present within the quartz (SiO_2_) mineral fraction [40]. Samples of sand and fine gravel (granules) from the el Forn and la Mina profiles (Figure 2) were dried, crushed, and sieved to 0.25–1 mm. The purified quartz obtained from each sample by selective chemical dissolution [41] was then decontaminated by removing the atmospheric component through sequential dissolutions [42]. The pure decontaminated quartz was then spiked with ∼100 µl of an in-house 3.10^−3^ g/g ^9^Be carrier solution prepared from deep-mined phenakite [43], in order to obtain a ponderable quantity of matter and to fix the ^10^Be/^9^Be value before performing any chemical processes. It was finally totally dissolved in concentrated HF. After replacing HF with HNO_3_, an aliquot of 500 µl of obtained solution was taken for measuring the concentration of ^27^Al. Al and Be were separated from the remaining solution by ion exchange chromatography and selective precipitation [43]. The resulting Be and Al oxyhydroxides were oxidized by heating at 800°C for an hour and the oxides prepared for Accelerator Mass Spectrometry (AMS) measurements. All the data reported in this study was measured at the French national facility ASTER (CEREGE, Aix-en-Provence). ^10^Be concentrations were normalized to ^10^Be/^9^Be SRM 4325 NIST standard with an assigned value of (2.79±0.03)×10^−11^. This standardization is equivalent to 07KNSTD within rounding error. The ^26^Al/^27^Al ratios obtained were calibrated against the ASTER in-house standard SM-Al-11 with ^26^Al/^27^Al = (7.401±0.064)×10^−12^ which had been cross-calibrated against the primary standards certified by a round-robin exercise [44] and using a ^26^Al half-life of (7.17±0.17)×10^5^ years [45], [46]. ^27^Al concentrations, naturally present in the samples, were measured at CEREGE by ICP-OES. Analytical uncertainties (reported as 1σ) include uncertainties associated with AMS counting statistics, AMS internal error (0.5%), chemical blank measurement, and, regarding ^26^Al, ^27^Al measurement. Long term measurements of chemically processed blank yielded ratios in the order of (3.0±1.5)×10^−15^ for ^10^Be and (2.2±2.0)×10^−15^ for ^26^Al [47].

When measured against ^26^Al KNSTD10650, the standard ASTER could be replaced by one that was available and had been cross-calibrated against the primary standards certified by a round-robin exercise ^26^Al, yielded a ratio of 7.554±0.104 ×10^−12^, 2.1% higher than the nominal value (CRONUS calculator documentation, 2009, page 6). The SM-Al-11/07KNSTD standardization used here therefore implies a ^26^Al/^10^Be production ratio of 6.61±0.50. This value and its associated uncertainty are directly derived from the update of the ratio originally determined by Nishiizumi et al. [48]. This production ratio was used in all calculations and modelling for ^26^Al.

Burial ages and denudation rates were calculated following the model fully described in the supplementary online material of Pappu et al. [18] that used the parameters discussed in Braucher et al. [49], including the ^26^Al/^10^Be production ratio of 6.61±0.50. The burial dating method makes it possible to determine the duration of burial episodes lasting from 100 ka up to 5 Ma [40]. This method is based on the differential radioactive decay of the two cosmogenic nuclides ^26^Al (T1/2 = 0.717±0.017 Ma) [45], [46] and ^10^Be (T1/2 = 1.387±0.012 Ma) [50], [51]. Neutron production rates were scaled using [52], and are based on a production ratio at sea level and high latitude of 4.49±0.30 at.g^−1^.a^−1^ for the ^10^Be.

## Results

### Palaeomagnetism

Several palaeomagnetic samples presented very low natural remanent magnetization (NRM) intensities or unstable demagnetizations, which made isolating the characteristic palaeomagnetic component impossible. However, 42 samples of the 108 exhibited a stable NRM during demagnetization, yielding a characteristic component with a consistent polarity sequence interpreted as representative of the Earth’s magnetic field. Magnetic behaviour varies between the different units and lithologies of the profiles measured; however, the magnetization of a large proportion of samples with a stable characteristic component is mainly due to magnetite and/or hematite.

**Figure 3:**
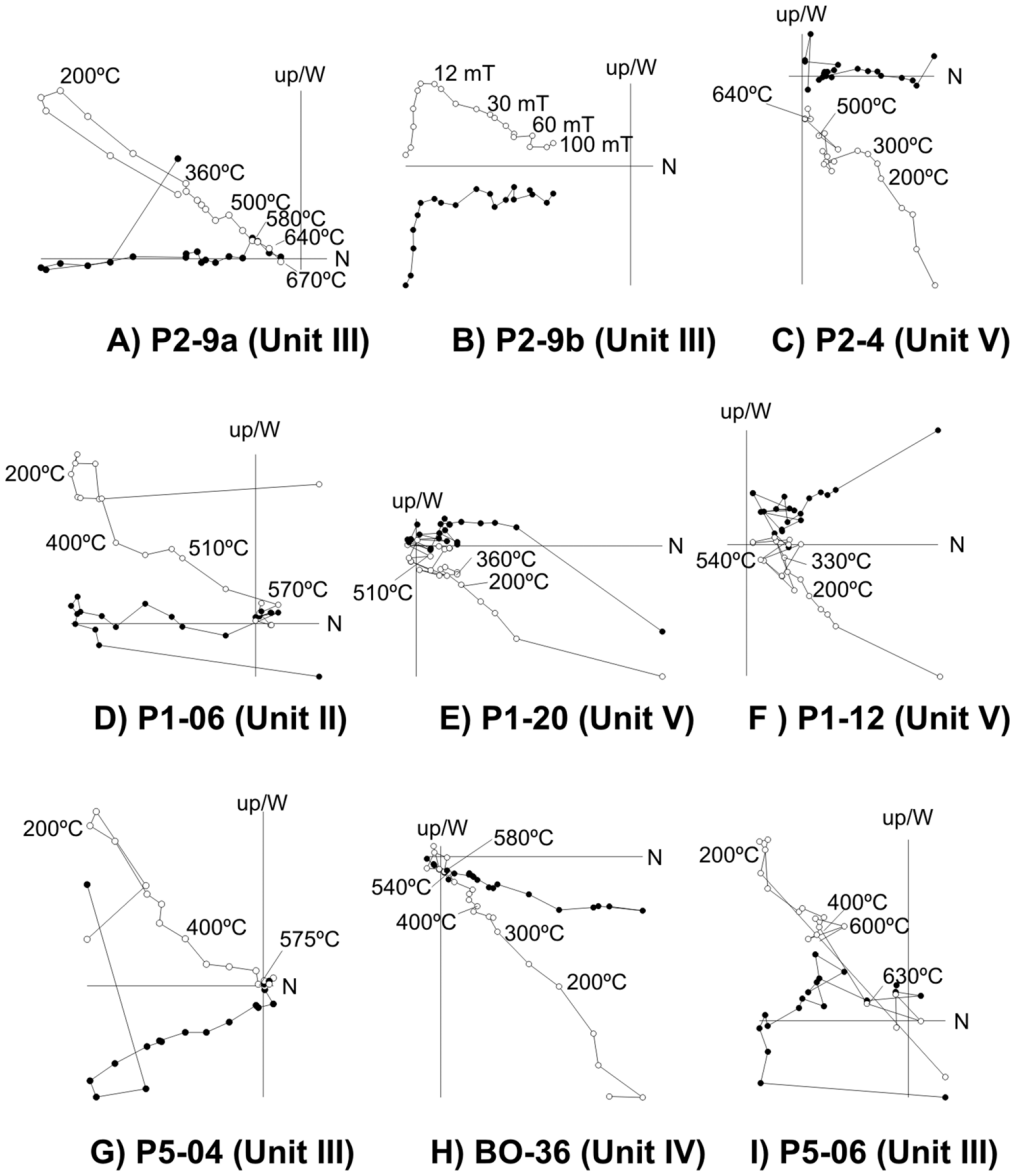
Orthogonal NRM demagnetization plots for representative thermal (A, C to -I) and alternating (B) field demagnetized samples. Directions are projected in geographic coordinates. Solid (open) points indicate projections of vector endpoints onto the horizontal (vertical) plane N-S. Sample codes and stratigraphic units are also indicated.

**Figure 4:**
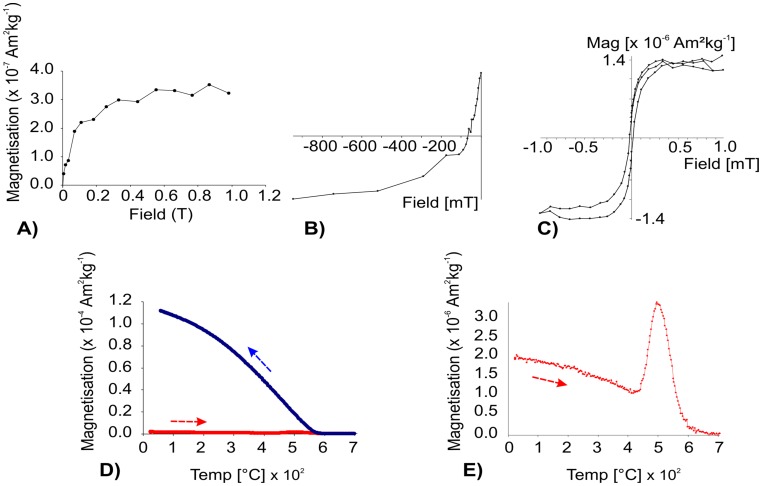
Rock magnetic experiments conducted on a representative sample (P2-9), corresponding to Zijdervelt diagrams of the Figure 3A and B. A) Progressive isothermal remanent magnetization (IRM) acquisition curve. B) Back-field curve. C) Hysteresis loop (±1 T). D) Thermomagnetic curve; arrows indicate heating and cooling cycles. E) Heating cycle of the Figure 4 D was amplified.

In a large number of samples, the characteristic component was isolated after removing a viscous component by heating up to 200°C or applying a peak alternating field of 12 mT (Figures 3 A, B, D, G, H and I). The maximum unblocking temperature for this component varied. In some cases it was close to the Curie temperature of magnetite (Figures 3 G and H). In others, the unblocking temperature spectrum reached temperatures of over 600°C, indicating that hematite was involved in the magnetization (Figures 3 A, C and I). The demagnetization diagrams represented in Figures 3 A and 4 B correspond to the thermal and AF demagnetization of two samples from the same core. In both cases the ChRM shows a clear reverse polarity. AF demagnetization (Figure 3 B) indicates that this directional component is carried by low-intermediate coercivity (magnetite and/or maghemite) and high coercivity (hematite) mineral phases. Thermal demagnetization (Figure 3 A) indicated that the ChRM had unblocking temperatures of over 640°C, confirming the contribution of hematite. Occasionally, a large part of the NRM (70%) was destroyed at temperatures around 300°C (Figures 3 C, E and F). A cluster of directions defining a clear polarity was seen above this temperature.

We conducted rock magnetic experiments on a set of representative samples. Figure 4 illustrates the results of the various experiments performed on sample P2-9, which corresponds to the core of the NRM demagnetization examples shown in Figures 4 A and B. Progressive IRM acquisition curves (Figure 4 A) indicate that low-coercivity ferromagnetic minerals are saturated up to low fields (100 mT), although the sample was not completely saturated up to 1 T (maximum applied field), indicating the presence of high-coercivity minerals (e.g. hematite) as well. This observation was also confirmed by the back-field experiment (Figure 4 B). Likewise, the hysteresis loop – expressed on a mass-specific basis and corrected by the paramagnetic fraction – shows the wasp-waisted shape characteristic of a mixture of minerals with different coercivities [53]. The heating cycle of the thermomagnetic curve (Figure 4 E) suggests a magnetization reduction range typical of magnetite. However, the massive creation of magnetite during heating was most probably due to changes to pre-existing paramagnetic minerals, and thus masks the Curie temperatures of the original minerals. On cooling, secondary magnetite was created at high temperatures.

**Figure 5:**
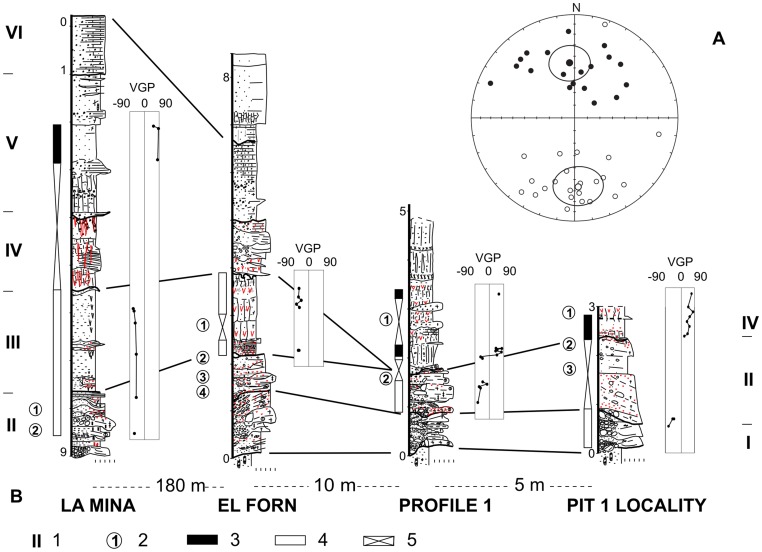
Reliable palaeomagnetic samples, with normal and reverse polarities, in the two antipodal hemispheres of the confidence circle and in the lithostratigraphic units of the Barranc de la Boella localities showing correlation lines. **A**. Equal-area projection of palaeomagnetic directions and characteristic component of the Barranc de la Boella samples with stable behaviour. Solid circles are projections in the lower hemisphere (normal polarity) and white circles are projections in the upper hemisphere (reverse polarity). In spite of the data dispersion, two mean directions corresponding to the samples with normal and reverse polarities can be observed. Both mean directions are antipodal considering alpha-95 confidence circle (positive reversal test). **B**. Correlations between the Barranc de la Boella stratigraphic units based on magnetostratigraphic and lithostratigraphic measures and observations. The magnetic measures are represented with the latitudes of the virtual geomagnetic pole (VGP) corresponding to the palaeomagnetic directions of the characteristic component. At all four outcrops we were able to define two magnetozones. A significant gap without representation due to a lack of reliable palaeomagnetic record in the samples is especially clear in Pit 1 Locality unit II. Keys: 1, lithostratigraphic units; 2, archaeopalaeontological levels; 3, normal polarity; 4, reverse polarity; 5, no palaeomagnetic record.

From the palaeomagnetic data reported here, we can conclude that the upper stratigraphic units (IV to VI) have normal polarity while the lower units (I to III) have reverse polarity (Figure 5). Fine-grained unit III is well represented in the EF and LM sections and contains a good palaeomagnetic record with samples showing only reverse polarity (Figure 5). However, reverse polarity in the unit III is not well represented in some sections due to the considerable discontinuity caused by hiatus. For example, in Pit 1 Locality is completely absent due to a hiatus with erosion resulting in a stratigraphic gap (Figure 5).

Profile 1 (P1) shows a clear consistent succession of two samples with reverse polarity and six samples of normal polarity within a muddy bed located at the base of unit IV (Figure 5). This transition has not been recorded in the Pit 1 Locality samples, as previously mentioned, because the basal part of unit IV is absent in this profile. The palaeomagnetic results therefore indicate that a polarity transition is located at the base of unit IV at P1 profile (Figure 5).

### Cosmogenic nuclides

All dates measured are minima and assume total shielding of the sample from cosmic rays (Table 3). However, at a depth of 9 meters, post-production by energetic particles can produce cosmogenic nuclides after the burial event. In that case, the nuclide concentrations measured are the sum of the inherited nuclides at the time of deposition, corrected for radioactive decay, and of the concentration produced at a constant depth since burial. This yields maximum burial ages (Table 3). The denudation rate, after the burial event, may be estimated to be 15 m/Ma.

**Table 3:** Barranc de la Boella radiometric dates obtained from sedimentary samples from stratigraphic units I and II at el Forn (EF) and la Mina (LM) localities (Figure 2).

Units	Samples	Depth (m)	^10^Be (at/g)	^26^Al (at/g)	Minimum burial age^a^ (Ma)	Denudation before burial (m/Ma)	Maximum burial age^b^ (Ma)
II	LM-1-Ru	9.12	71077±3580	32568±26558	0.87±0.08	31.09±2.98	1.20±0.11
II	LM-1-R	9.12	157996±5122	64204±27546	1.09±0.06	11.83±0.64	1.26±0.07
I-II	LM-2-R	7.87	71683±3341	187420±59827	2.05±0.66	16.52±5.33	3.87±1.25
II	EF-1-Ru	5.90	40590±3327	251553±18129	0.24±0.03	76.79±8.38	0.93±0.1
II	EF-1-R	5.90	86145±3651	37603±18501	0.97±0.06	24.15±1.57	1.53±0.1
II	EF-1-A	5.90	84164±3426	35403±20277	1.05±0.07	23.74±1.67	1.68±0.12
II	EF-2-A	6.10	94357±3911	39181±21855	1.07±0.07	20.78±1.44	1.60±0.11
I-II	EF-3-R	6.70	74650±3201	22015±16369	1.80±0.15	18.12±1.55	3.45±0.3
I	EF-4-R	7.30	74358±4031	18649±17236	2.14±0.23	15.18±1.63	4.41±0.47
I	EF-6-A	7.60	7696±4003	20809±13573	1.98±0.17	15.92±1.33	3.58±0.3
I	EF-5-R	8.20	20073±6381	38290±22720	2.60±0.18	3.92±0.26	3.22±0.22

La Mina sediment density = 1.52 g/cm^3^. El Forn sediment density = 1.46 g/cm^3^. The geographical parameters used for calculation are: latitude of 41.13587° for LM and 41.13475° for EF, an altitude of 50 m and a pressure of 1007 mbar. Neutronic production are 4.45 at.g^−1^.a^−1^ for ^10^Be and 29.40 at.g^−1^.a^−1^ for ^26^Al; slow muons production are 0.012 at.g^−1^.a^−1^ for ^10^Be and 0.861 at.g^−1^.a^−1^ for ^26^Al; and fast muons production are 0.039 at.g^−1^.a^−1^ for ^10^Be and 0.082 at.g^−1^.a^−1^ for ^26^Al [49].

a In the “minimum burial age” model, it is assumed that the samples did not accumulate cosmogenic nuclides while buried (infinite burial depth).

b In the “maximum burial age” model, it is assumed that the samples remained buried at their sampling depths and accumulated cosmogenic nuclides produced by muons. In this case, denudation rate is considered constant before and after burial (15±2 m/Ma). Burial age and denudation rate uncertainties (reported as 1σ) propagate the half-lives uncertainties.

The unit I samples, obtained in the EF locality, have minimum radiometric dates between 2.60±0.18 Ma (EF-5-R) and 1.80±0.15 Ma (EF-3-R) (Table 3). The average of the two radiometric dates of the upper half of unit I was 2.00±0.19 Ma (EF-3-R and EF-4-R) (Table 3). Measurement of the set of chronostratigraphical horizons between units I and II near the LM pit locality yielded a minimum low-precision date of 2.05±0.66 Ma (LM-2-R) (Table 3).

The radiometric dates measured in the unit II samples cover the time span between 0.87±0.08 Ma (LM-1-R) and 1.07±0.07 Ma (EF-2-A) (Table 3). Sample EF-1-Ru was dated to 0.24±0.03 Ma, but this date is clearly inaccurate given the available biochronological and palaeomagnetic evidence obtained for unit II. The dates provided by the remaining five samples from unit II support the lithostratigraphic correlation between the LM and EF localities and indicate a precise radiometric averaged minimum date of 1.00±0.068 Ma.

### Lithic assemblages

**Figure 6:**
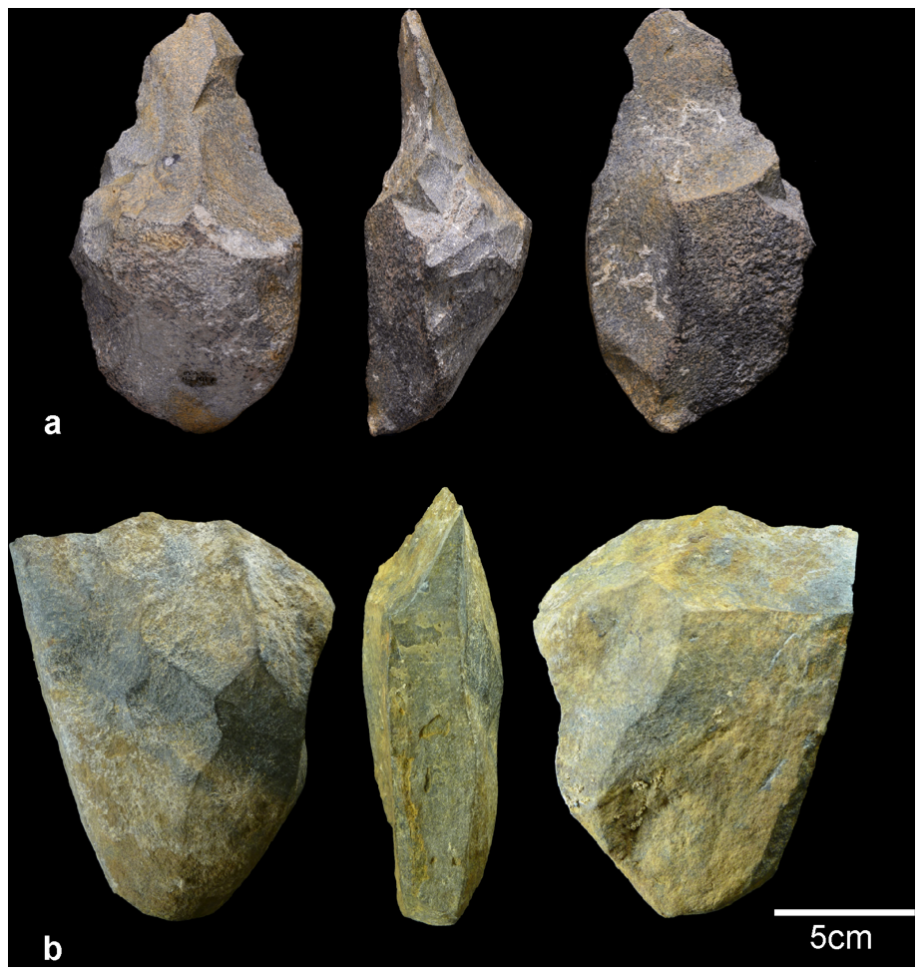
Barranc de la Boella large cutting tools (LCT). a) split cobble of schist showing trihedral pick configuration from level 2 at Pit 1 Locality (P1L) site; b) schist cleaver from level 2 at EF site.

The most significant lithic assemblage comes from P1L level 2 (n = 125) (Table 2). Most of the pieces here were made from chert, although schist, quartz, sandstone, granite and quartzite were also used, all of them locally available. This assemblage is made up of three hammerstones and seven fractured pebbles and fractured pebbles; three cores reflecting unipolar and occasionally centripetal flaking; 45 flakes, 37 broken flakes and flake fragments and 21 angular fragments. There are only eight retouched flakes, all of them notches and denticulates. Finally, one large tool was recovered: a pick made from a very thick schist flake (a split cobble) (Figure 6a). Around 17% of the flakes are less than 20 mm long. 11 refit groups were found which supports the integrity of the lithic assemblage.

The EF unit II (levels 2, 3 and 4) assemblage is composed of 104 items, including three hammerstone; 11pebbles and fractured pebbles mainly made from schist and one unipolar quartz core. The group of artefacts made from chert include seven cores, 46 flakes, 14 broken flakes and six retouched flakes (denticulates). The assemblage also contains one cleaver made from a massive schist flake (split cobble) (Figure 6b), one quartzite chopping tool, two choppers, one possible handaxe made from schist and one small bec made from granite.

Finally, the assemblage recovered from LM unit II (levels 1, 2 and 3) consists of 79 items, including two hammerstones made from sandstone and quartz, and nine fractured pebbles made from different raw materials: three schist choppers and one porphyry chopping tool; and five scarcely knapped chert cores. The products of knapping on chert include 25 flakes, 21 broken flakes and flake fragments, and seven retouched flakes (mainly notches and denticulates).

**Figure 7:**
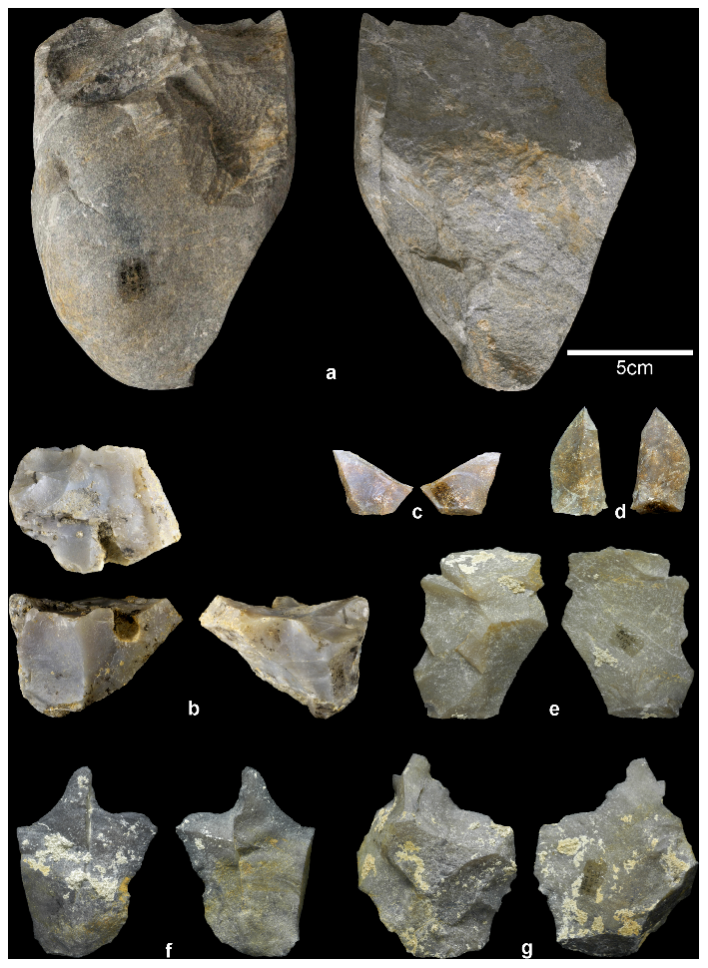
Barranc de la Boella cores, flakes and retouched tools. a) Big schist core with longitudinal flaking from level 2 at P1L site; b) core on chert scarcely exploited from level 1 at EF site; c and d) small chert flakes from level 2 at P1L site; e and f) medium-sized chert flakes from level 2 at P1L site; g) middle-sized chert retouched flake –denticulate- from level 2 at P1L site.

A preliminary technological diagnosis of the P1L and EF localities lithic assemblages points to early Acheulian technology. The components of Acheulian represented in the Barranc de la Boella lithic industry consist of few large cutting tools (LCT) (Figure 6) and small and medium-sized flakes and retouched tools made from chert (Figure 7). The split cobble technique is present in the North African Oldowan record (e.g. Monts Tessala) [54], but it is generally currently considered a defining characteristic of the early Acheulian of the African continent [1], [12], [55].

### Faunal assemblages

The main faunal assemblage of the Barranc de la Boella record comes from the level 2 at P1L, which contains skeletal elements of one individual of *Mammuthus meridionalis* (NISP = 485), but to date only 160 anatomical elements have been identified. Other faunal remains found in level 2 belong to Cervidae indet. (n = 4), a neonatal tooth fragment from *Mammuthus meridionalis* (n = 1) and seven bone fragments belonging to from animals in weight class 3 [56].

The *M. meridionalis* remains were mainly from the cranial skeleton (abundant skull fragments and all the teeth); along with these, two scapulae were recovered and several rib fragments and three vertebrae (one cervical vertebra and two fragments of indeterminate vertebrae). The tusks and molars indicate that this was a young adult individual that died at the age of about 30. The molars and one of the tusks were recovered in the anatomical position. The remaining pieces were grouped but disjointed and distributed around one of the tusks. The spatial distribution of the faunal remains shows a clustering of skull fragments around the teeth and a similar distribution of the rib fragments. One of the scapulae was very fragmented when found, although its remains were clustered together.

This high degree of fracturing may have occurred as a result of trampling by other Proboscidea, as suggested by the abundant notches in the edge of the fractures [57]; the other scapula has survived almost intact. The fragmentation of the ribs, skull and one of the scapulae may be also the result of trampling by other mammoths. The cranial fragments and the left scapula display little spatial dispersion, indicating that fragmentation occurred in primary position. One of the mammoth tusks has probably moved vertically, based on its location in the mud of unit IV. The postdepositional modifications responsible for the vertical position of some of the bones that were identified in the faunal record of level 2 may also be attributable to the mechanical process which forms fissures in sediments, analogous to the behaviour of vertic B soil-horizons, described in the muddy units III, IV and V of the Barranc de la Boella lithostratigraphy.

At Level 3 P1L other remains of *M. meridionalis* composed of teeth (2 tusks, 4 molars) and skull fragments were found.

The taxonomic variety in LM (n = 714) is higher (Table 1). These remains from this locality are highly fractured. Only 1.1% of the bones are complete. These are the phalanges and metapodials of animals of various weights (from Leporidae to *Equus* sp.) and the radii and ulnas of Cervidae. This assemblage is made up of elements of differing sizes (ranging from 2 to 250cm long) which were found scattered around the excavation area. The presence, in a cluster, of several remains of an *Ursus* sp. forelimb at level 2, indicates that burial was rapid and at least some of the remains had not been significantly moved. In this case, the specimens are better conserved than in the case of the P1L assemblage. However, albeit to a lesser extent, they are have also been affected by chemical changes caused by sediment leaching, which made it impossible to clearly identify any cut marks on the bone surfaces.

Carnivorous activity has been identified through tooth marks, the fact that the epiphyses of long bones had been eaten, and the presence of large bone fragments with evidence of digestion. Of the residues, 7.7% show any of these modifications. Carnivorous consumption occurred in some cases, as shown by the scooping-out of long bones of large animals such as *Equus* sp. and Bovini. These modifications, along with the presence of coprolites, suggest the presence of a large hyena, perhaps *Pachycrocuta brevirostris*, although no skeletal remains of these animals have been found at this site. We have, however, identified a fragment of a premolar cusp in level III assemblage from EF.

In EF, 796 faunal remains were recovered. The taxonomic diversity is high and similar to LM, although some of the LM taxa are absent and others, such as *Sus strozzi*, have been identified (Table 1). These assemblages principally include those elements most likely to survive (the teeth and fragments of long bone shafts). In general, the degree of change suffered by the remains is greater than at the other two locations excavated.

## Discussion

The biochronological data indicate that the reverse polarity documented in units II and III corresponds to the Matuyama chron. The Brunhes/Matuyama polarity transition was recorded at the base of unit IV. The Brunhes chron has been identified in the upper part of unit IV in P1 and in unit V in the LM locality (Figure 5). A reliable geochronological age for the lithic assemblages found within Barranc de la Boella unit II, constrained by biostratigraphy and magnetostratigraphy, indicate late Matuyama chron (0.96–0.78 Ma) and late Early Pleistocene Sub-epoch/Sub-series of the international geological time scale.

The Barranc de la Boella site has yielded a set of LCT lithic assemblages in unit II that suggests that the Acheulian technological tradition was present in Europe before 0.78 Ma. As mentioned in section 4.3, lithic artefacts represented in the Barranc de la Boella record constitute an assemblage of few LCT, flakes and retouched tools, but not an isolated findings of single artefacts (Figures 6 and 7) [30]–[32], [58]. The fossil record in the archaeopalaeontological levels of the Barranc de la Boella suggests different sedimentary and behavioural contexts (Figure 5). The fossil assemblage of level 2 of the P1L excavation has great potential to illustrate a prehistoric activity area and provide insight into land use strategies [59]. The stone tools are found around the remains of *Mammuthus meridionalis*. The composition of the fossil assemblage documented in level 2 of P1L may be related, according to Leakey’s classification [21], to the butchering site of a single large herbivore. In contrast, our observations at several levels of LM and level 3 of EF and P1L suggest findings in secondary position, located within fluvial and debris flow deposits, indicating some similarity to the sites located in the vicinity and within flowing streams. One isolated schist cleaver found in EF level 2 is associated with dispersed bone remains. These archaeopalaeontological assemblages may be considered analogous to the sites with diffuse material also described by Leakey [21].

The fossil assemblages of Barranc de la Boella were probably exhumed from a past flooded habitat near the confluence of a tributary and the axial river at the lower valley of the Francolí River basin. Hominins discarded the lithic remains after accessing carcasses of large animals trapped or killed in channels and pools of water, as documented in present-day studies [57], [60]. Single, large herbivore butchery sites have been described as part of the daily forager range of modern hunter-gatherers in accordance with land use based on home bases or central sites [59], [61]. This analogy suggests that the Barranc de la Boella hominins were meat eaters, implying a high trophic level in these late Early Pleistocene European ecosystems.

**Figure 8:**
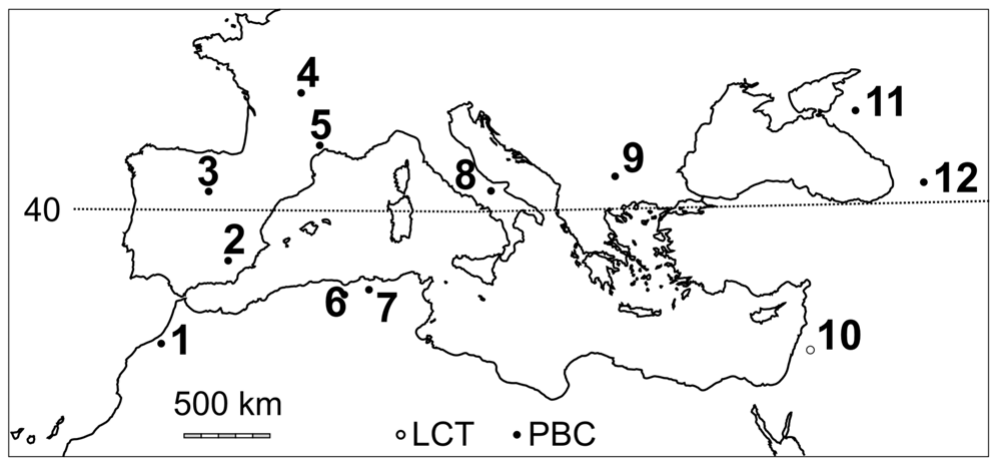
Lithic assemblages in the archaeological sites around the Mediterranean Sea between 2 and 1 Ma ago. LCT: Large Cutting Tools technology; PBC: Pebble and Core technology [14]. Legend: 1, Casablanca sites, Morocco; 2, Orce sites, Spain; 3, Sima el Elefante, Spain; 4, Pont de Lavaud, Lunery, France; 5, Lézignan le Cèbe, France; 6, 1. El Kherba and Ain Hanech, Algeria; 7, Mansourah, Algeria, 8, Pirro Nord, Italy; 9, Kozarnika, Bulgaria; 10, Ubeidiya, Israel; 11, Rodniki, Bogatyri, Russia; 12, Dmanisi, Georgia.

**Figure 9:**
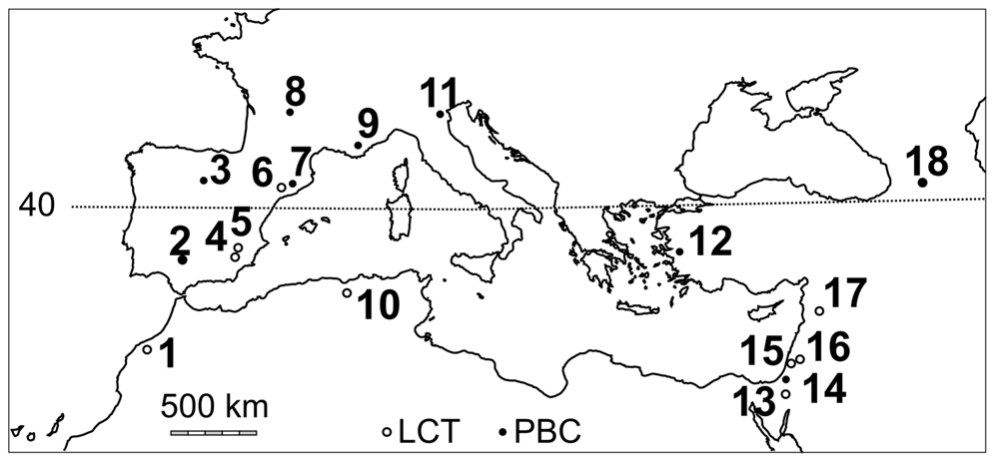
Lithic assemblages in the archaeological sites around the Mediterranean Sea between 1 and 0.7 Ma ago. LCT: Large Cutting Tools technology; PBC: Pebble and Core technology [14]. Legend: 1, Thomas Quarry I, Morocco; 2. Guadalquivir, Spain; 3, Gran Dolina, Spain; 4, Cueva Negra, Spain; 5, Solana del Zamborino, Spain; 7, Vallparadís, Spain; 6, Barranc de la Boella, Spain; 8, La Noira, France; 9, Vallonet, France; 10, Tighenif and Erraya, Algeria; 11, Monte Poggiolo, Italy; 12, Dursunlu, Turkey; 13, Evron, Israel; 14, Nahal Zihor, Israel; 15, Bizat Rumana, Israel; 16, Gesher Benot Yaaqov, Israel; 17, Latame, Syria; 18, Ahkalkalaki, Georgia.

In the past quarter century, increasing numbers of lithic assemblages have been exhumed at prehistoric sites on the Iberian Peninsula. These findings have fuelled the discussion about the taxonomic classification of the prehistoric cultural repertoires in Eurasia (see section 1) [7], [62]. The density of prehistoric sites dated to before 1 Ma clustered around the Mediterranean, not only indicates the early dates of archaeological sites with PBC technologies, but is of the same order of magnitude as those sites identified as falling within the EMPT time interval of 1 to 0.7 Ma that have LCT and PBC technologies (Figures 8 and 9). In the case of the Italian Peninsula, the density is one location every 100 ka for the 1 to 0.7 Ma range [63]. On the Iberian Peninsula (Table 4), the density is slightly higher, with two locations per 100 ka for the 1 to 0.7 Ma range, although the quality of the raw data shows an uneven distribution, as exemplified by the Cúllar-Baza 1, Solana del Zamborino, Cueva Negra and Vallparadís lithic assemblages [30], [32], [64], [65]. Meanwhile, the best primary data to describe human settlement over time in the EMPT era has come from the Gran Dolina cave record [66], [67]. Human occupation of the Atapuerca sites during the EMPT (Gran Dolina, Sima de los Elefantes), along with other evidence from Western Europe, suggests that there was a discontinuity in the human occupation of Europe which occurred around the Matuyama-Brunhes boundary.

**Table 4:** Primary data from Iberian archaeological sites dating in the Early-Middle Pleistocene Transition time span (942 to 641 kyr).

Site complex and archaeological level	Fluvial basin	Biozone^a^	Sedimentary setting	Lithic assemblages^b^
Vallparadís 10	Llobregat	MQ3	Alluvial	PBC
Atapuerca TD4	Duero	MQ3	Cave entrance	PBC
Atapuerca TD6	Duero	MQ4	Cave entrance	PBC
Boella Unit II	Francolí	MQ4	Alluvial	LCT
Cúllar – Baza 1	Baza	MQ4	Alluvial	LCT
Cueva Negra	Segura	MQ4	Cave entrance	LCT

a MQ, Iberian biozones. ^b^LCT: large cutting technology; PBC: pebble and core technology [14].

The evidence for continuity or discontinuity in prehistoric settlement of the Iberian Peninsula during the EMPT has implications for the debate on how the early LCT innovation found in the European Palaeolithic record came about. The majority of LCT assemblages on the Iberian Peninsula and in Europe date to the end of the EMPT and later (<0.7 Ma) [10], [31]. Evidence of this has been claimed at Atapuerca as well as by regional archaeological research conducted in Iberian terrace sequences (Table 5). In Table 5, we summarize the scant evidence of lithic assemblages in the Iberian fluvial terrace complexes with elevations above or equal to +40 m, although it is true that not all sites contain deeply stratified lithic assemblages (e.g. Puig d’en Roca) [68]. Iberian LCT lithic assemblages have been found at prehistoric sites set in Middle Pleistocene age river terraces (T +20 to T +30 m), which is where the most significant Iberian Acheulian sites have been found (Table 5) [6], [7], [69].

**Table 5:** Summary of archaeological sites in terraces and their elevations (>30 m) related to archaeological assemblages or single potential artefacts of the Iberian fluvial basins dating roughly in the end of Early-Middle Pleistocene Transition (0.78–0.5 Ma).

Site complex and Fluvial Terrace Elevation	River basin	Site size	Lithic assemblages^a^
Ambrona T+40 m	Duero	dm^2^	LCT
Cansaladeta T+40 m	Francolí	m^2^	LCT
Guadalquivir T 40–60 m	Guadalquivir	m^2^	PBC and LCT
Pinedo T+30–40/+75–80 m	Tajo	m^2^	LCT
Maya T+30–50 m	Duero	m^2^	LCT
Puente Pino T+30–50 m	Tajo	m^2^	LCT
Puig d’en Roca T+50–80 m	Ter	m^2^	PBC and LCT
Sartalejo T+20–40 m	Tajo	m^2^	LCT
Yeltes Huerba T+30–50 m	Duero	m^2^	LCT

In this summary we note scarce representation of pebble and core artefact assemblages. These terrace systems contains, in the lower and middle elevations (<30 m), the archaeological sites of Middle Pleistocene age with LCT artefact assemblages of Iberian Peninsula record (various sources) dating to ∼<500 kyrs described in the right column of the table.

a LCT: technology; PBC: technology [14].

The Barranc de la Boella deposits are incised in T +60 m and stratified with the T +50 m terrace of the Francolí River valley. These elevations constitute an exception in terrace sequences in the Iberian record. Only a single potential artefact has previously been reported on the high terraces of the Iberian fluvial basins [70]. Recently, new archaeological sites featuring lithic assemblages with LCT have begun to emerge in the high terraces of the Tagus [71]. Discontinuous prehistoric settlements, established in territories characterised by populations undergoing biological dispersion [72], may explain the small amount of primary data in Early Pleistocene fluvial deposits on the Iberian peninsula. Conversely, Bridgland points to the chronological inconsistency in the Iberian Palaeolithic record, between the Early Pleistocene age of the Lower Palaeolithic in Atapuerca, Orce and other archaeological sites found in terrace sequences and caves in the rest of Europe [27], [73]–[75], and the Lower Palaeolithic record in the Iberian fluvial archives dated to the Middle Pleistocene [24]. A taphonomic bias that could explain the absence of a Lower Palaeolithic record in the Middle Pleistocene terraces of Mediterranean river basins and the presence of such a record at Iberian Atlantic basins, was excluded some years ago [76]. These temporal inconsistencies between the Lower Palaeolithic record of the Iberian terrace sequences and the early Lower Palaeolithic record in Europe seem to indicate that further geological (geochronology) and archaeological work is needed on the Iberian river basins [24], [77]. To sum up, two possibilities may explain the inconsistency between the geochronology of the first occupations at Atapuerca and Orce (Early Pleistocene) and the regional Archaeology of the Iberian terrace sequences dated to the Middle Pleistocene: the lack of more systematic research conducted in river basins (terraces >30 m elevation); and/or that successive and separate hominin dispersals depopulated this part of Europe, which acted like a biogeographic sink-hole during EMPT [78].

## Conclusions

The archaeological record of Barranc de la Boella confirms the biogeographic distribution of earliest LCT assemblages across southern Eurasia during the late Early Pleistocene (Figure 1). Up to now, the date for the earliest Acheulian LCT technology matched hominin colonization of the subcontinent and was placed at the start of the Middle Pleistocene, later than that described for North Africa and Asia [10], [11], [18], [79]–[82].

The chronology of the LCT assemblage found at Barranc de la Boella indicates that the Acheulian technical tradition first appeared in Europe around 0.96–0.781 Ma ago. The early European Acheulian assemblage has been found in areas populated by hominins during previous dispersal events with PBC technologies. The increase in technical behavioural diversity has been found in the huge expanse of geographical, temporal and ecological territories occupied by hominins [83]. It is thus reasonable to expect changes in land use and technical skills which, in our opinion, may be attributed to the chronology of various poorly recorded hominin dispersal events that date to before the definitive colonization of Eurasia during the Middle Pleistocene.

## Supporting Information

Figure S1 ***Mammuthus meridionalis*** **dental remains found at Barranc de la Boella in pit 1 locality level 2.** Left: upper M1 (BB07 C1 N2 O12 n° 84). Rigth is upper M3 (BB07 C1 N2 P13 n° 115). Both scale bar 5 cm.
(TIF)

Figure S2 A: M3, M1 and broken M1 from *M. savini* found from unit II in pit 2 or la Mina locality. B: Bucal, occlusal and labial views of m1 (top) and m2 (bottom) from *V. chalinei* sampled in unit II at pit 2 or la Mina site. C, Bucal, occlusal and labial views of m1 from *M. savini* (top two) and enamel remains of one m1 and two M3’s from M. *savini* (bottom, left to right) recorded in pit 3 or el Forn unit II. Scale bar 1 mm.
(TIF)

Figure S3 **Graphic representation comparing the length (L) and width (W) of the Barranc de la Boella sample and other documented** ***Mimomys savini*** **specimens from selected Iberian sites.** These Iberian sites are arranged from older (left) to younger (right) (except Barranc de la Boella).
(TIF)

Information S1 **The large mammals at Barranc de la Boella localities.**
(DOC)

Information S2 **Small mammals at Barranc de la Boella localities. Table S1**, Average, maximum and minimum values for the length (L) and width (W) of the *Mimomys savini* m1 from early Pleistocene Iberian sites.
(DOC)
