# Supplementary figures and images for: Age and Date for Early Arrival of the Acheulian in Europe (Barranc de la Boella, la Canonja, Spain)
Source: PLoS One. 2014 Jul 30;9(7):e103634. doi: 10.1371/journal.pone.0103634 (PMC4116235; doi:10.1371/journal.pone.0103634)

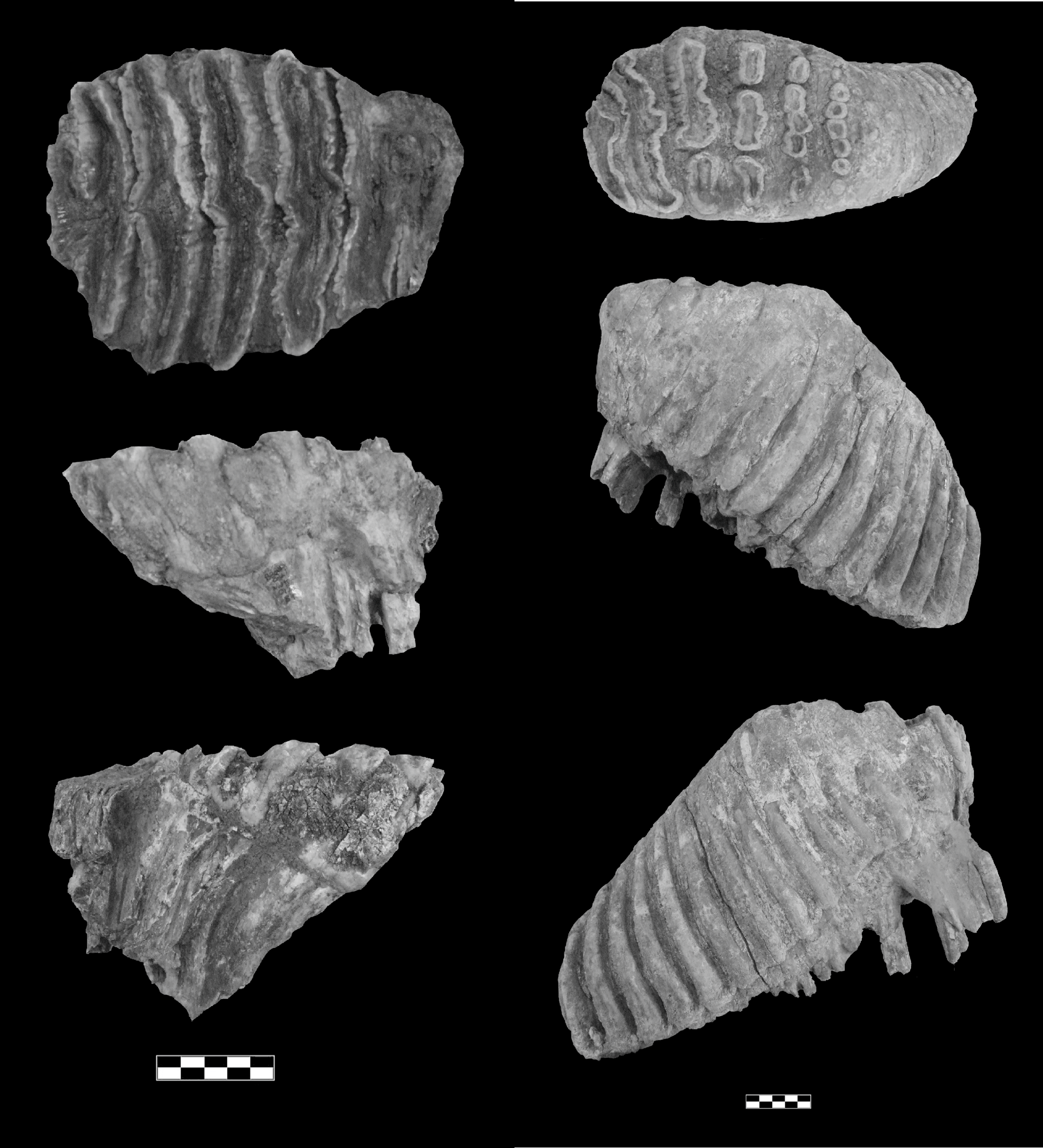

Supplement: Figure S1 — Mammuthus meridionalis dental remains found at Barranc de la Boella in pit 1 locality level 2. Left: upper M1 (BB07 C1 N2 O12 n° 84). Rigth is upper M3 (BB07 C1 N2 P13 n° 115). Both scale bar 5 cm. (TIF) [file pone.0103634.s001.tif]

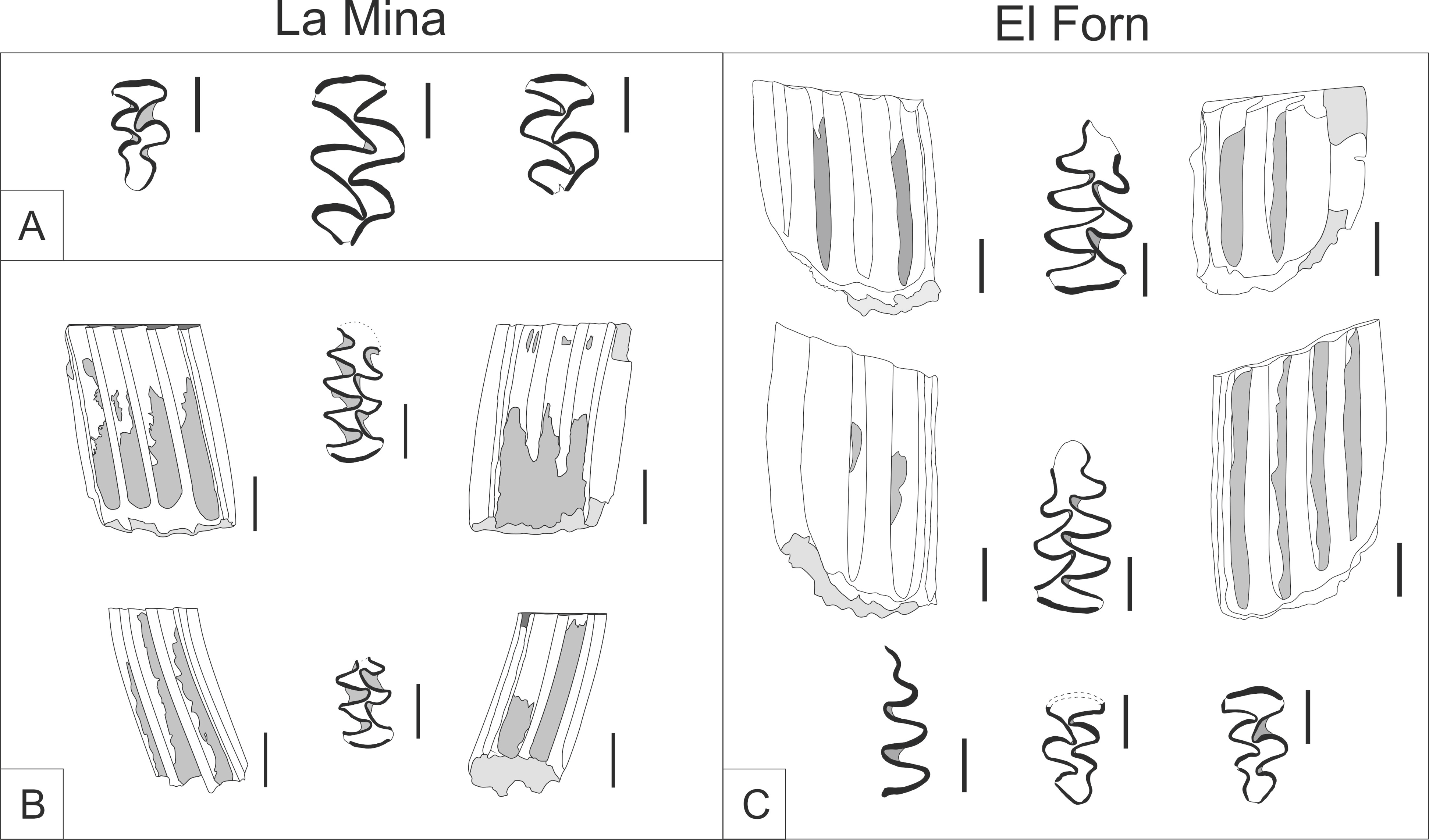

Supplement: Figure S2 — A: M3, M1 and broken M1 from M. savini found from unit II in pit 2 or la Mina locality. B: Bucal, occlusal and labial views of m1 (top) and m2 (bottom) from V. chalinei sampled in unit II at pit 2 or la Mina site. C, Bucal, occlusal and labial views of m1 from M. savini (top two) and enamel remains of one m1 and two M3’s from M. savini (bottom, left to right) recorded in pit 3 or el Forn unit II. Scale bar 1 mm. (TIF) [file pone.0103634.s002.tif]

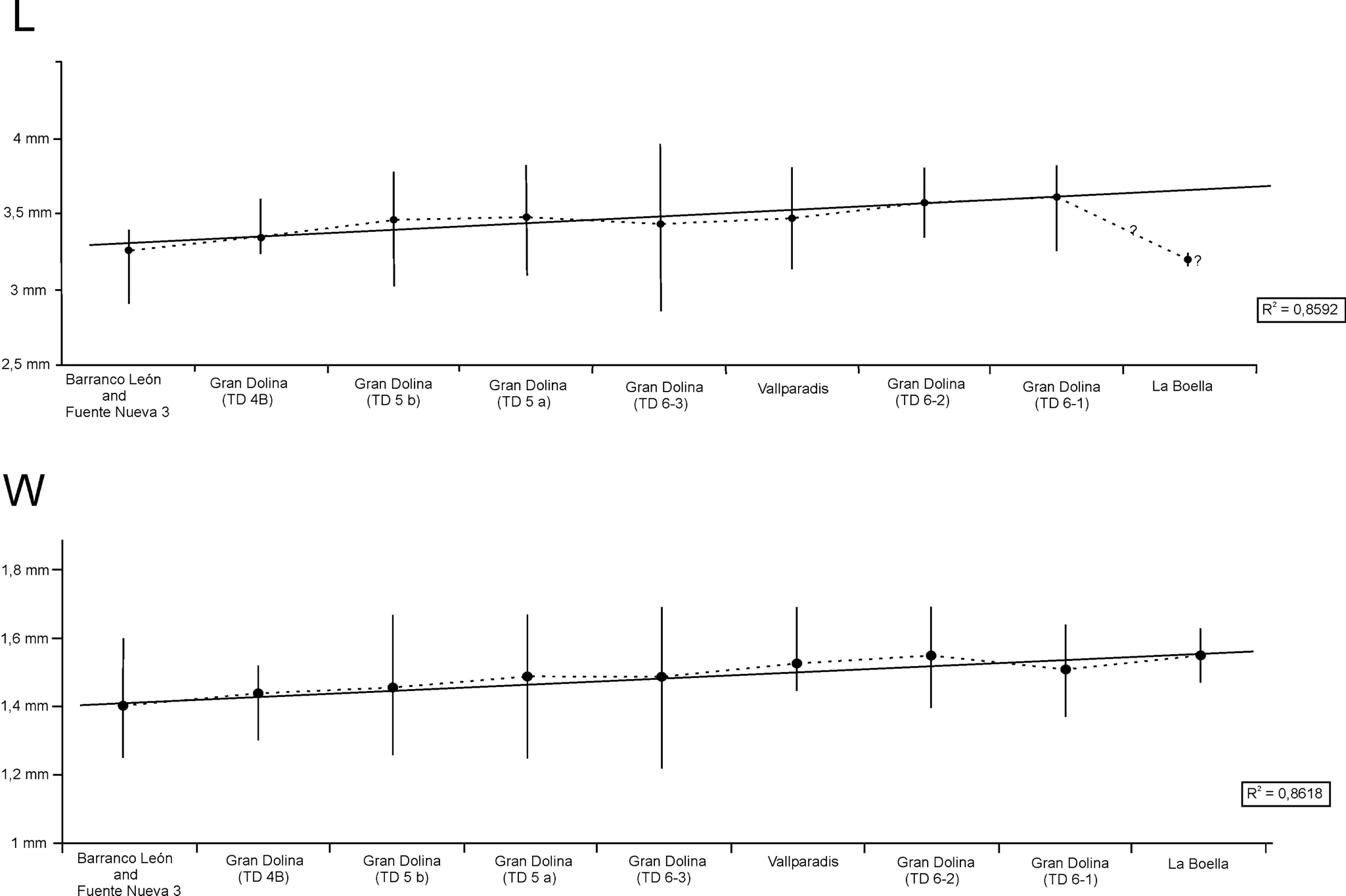

Supplement: Figure S3 — Graphic representation comparing the length (L) and width (W) of the Barranc de la Boella sample and other documented Mimomys savini specimens from selected Iberian sites. These Iberian sites are arranged from older (left) to younger (right) (except Barranc de la Boella). (TIF) [file pone.0103634.s003.tif]
