# Supplementary material for: Age and Date for Early Arrival of the Acheulian in Europe (Barranc de la Boella, la Canonja, Spain)
Source: PLoS One. 2014 Jul 30;9(7):e103634. doi: 10.1371/journal.pone.0103634 (PMC4116235; doi:10.1371/journal.pone.0103634)
Supplement: Information S1 — The large mammals at Barranc de la Boella localities. (DOC) [file pone.0103634.s004.doc]

**Supporting Information S1. The large mammals at Barranc de la Boella localities.**

*Introduction*

The site of Barranc de la Boella is characterized by the record of large fossil mammals dominated by the presence of megaherbivores, especially proboscideans (*Mammuthus meridionalis*) together with hippopotamids (*Hippopotamus antiquus*), rhinocerotids (*Stephanorhinus hundsheimensis*), and also by other smaller size herbivores, equids (*Equus* sp.) and the cervids [Cervidae indet. (large size) and Cervidae indet. (middle size]. The record of carnivores is scanty, only the ursid *Ursus* sp., the middle size canid *Canis mosbachensis*, together with several hyena coprolites (cf. *Pachycrocuta brevirostris*). This faunal assemblage corresponds to the latest Villafranchian [1,2], and this site is one of the youngest records with presence of these taxa.

*Systematic Palaeontology*

**Order PROBOSCIDEA Illiger, 1811**

Family ELEPHANTIDAE Gray, 1821

Genus *Mammuthus* Brookes, 1828

Species *Mammuthus* *meridionalis* (Nesti, 1825)

Material: 1 left first upper molar (M1) (BB07 C1 NIV 2 O12 nº 84) (Fig. C.1), 4 third upper molars (M3) (BB 07 C1 NIV 2 P-13 nº 57; BB 07 C1 NIV 2 P-13 nº 58; BB 07 C1 NIV 3 O-12 nº 4 and BB07 C1 NIV 2 P13 nº 115 (Figure S1), 45 isolated plate fragments, 4 mostly complete tusks and 12 isolated enamel fragments, 1 left scapula (BB07 C1 N2 O13 nº 126) and 2 rib fragments.

M1: It is a well-preserved complete specimen. It shows seven enamel plates, including both talons and the roots. It is worn, especially in the mesial region, and shows the typical occlusal figure of the genus *Mammuthus*. The cement is abundant; the enamel is thick and folded. The plate frequency is relatively high, five plates per ten cm, which is typical of the advanced forms of *Mammuthus meridionalis*.

M3: The 4 specimens are well-preserved and complete. As in the M1 specimen, all of them show the typical occlusal figure of the genus, plate frequency, enamel thickness and crown high corresponding to the characteristics of *Mammuthus meridionalis*. By the number of plates, 17 in the specimen BB 07 C1 N3 O-12 nº 4, we can see that it corresponds to an advanced form of the species.

Tusks: 4 mostly complete tusks have been recovered. All of them show strong twist, which is a typical character of the genus *Mammuthus*. We have done an enamel analysis of the Shreger lines, and the calculated angles are included in the variability of the genus *Mammuthus*. 12 more enamel fragments were also recovered.

Scapula: Only one scapula in bad preserved conditions has been found. It has the scapular spine. Both, supra and infraspinossus, fossae are incompletes, only preserving two third parts of them.

Ribs: The other postcraneal preserved specimens are rib fragments.

Comments: By the anatomy of the dentition, we can say that the *Mammunthus* *meridionalis* specimens recorded at Barranc de la Boella correspond to an advanced-terminal form, with high plate frequency, low average enamel thickness, and high number of plates in the third molars.

**Order ARTIODACTYLA Owen, 1848**

Family HIPPOPOTAMIDAE (Gray, 1821)

Genus *Hippopotamus* (Linnaeus, 1758)

Species *Hippopotamus antiquus* (Desmarest, 1822)

Material: Right palate with P3-M3 (BB09 LA MINA C2 NIV 2 S15 nº 15); left palate with P3-M2 (BB09 LA MINA C2 NIV 2 S15 nº 22); Canine fragment (BB09 C2 NIV 2 M14 nº 5); right upper third premolar (P3) (BB08 C2 NIV 2 P14 nº19); cranial fragment (BB09 C2 NIV 2 R14 nº 3) and left calcaneum fragment (BB08 C3 NIV 2 H13 nº 1).

Six specimens corresponding to this amphibian megaherbivore species have been recovered until now, a mostly complete maxilar with the right series P3-M3 and the left one P3-M2, a canine fragment, a posterior cranial fragment, and only one postcraneal piece, a left medial calcaneum fragment.

The comparative metric and morphologic analyses of the teeth from Barranc de la Boella, together with those from the European sites of Fuente Nueva 3, Barranco León, Huéscar 1, and Cal Guardiola (Spain) [3-5], and Upper Valdarno (Italy) [6] corresponding to *Hippopotamus antiquus*, those from 'Ubeidiya (Israel), ascribed to *Hippopotamus gorgops* and *H. Behemoth* [7], those from Buia (Eritrea) corresponding to *H. gorgops* [8], and those from the extant species H. amphibius, show that the specimens from Barranc de la Boella fall in the variability of *H. antiquus*.

Comments: *Hippopotamus antiquus* is an African origin species that arrives into Europe during the Late Villafranchian and becomes extinct at the base of the middle Pleistocene, during the Galerian.

Family CERVIDAE Gray, 1821

Subfamily CERVINAE Baird, 1961

Species Cervidae indet. (middle size)

Material: M1 ó M2 (BB09 C3 N1 M11 nº 4); M3 (BB09 C3 N1 N11 n º 1); I (BB08 C2 N2I R14 SIN Nº); I (BB08 C2 N2 R15 nº 3); left M1 (BB08 C2 N2 Q24 nº 15, BOLSA DE NIVEL) and two antler fragments (BBO9 C3 N1 O14 nº10 and BB09 C3 N1 O14 nº 6).

Seven specimens corresponding to a deer middle size species Cervidae indet. (middle size) have been recovered, five of them are isolated teeth and the other two correspond to antler fragments. The comparison of the metric data of the first lower molar with those from Barranco León and Fuente Nueva-3 (Spain) ascribed to *Metacervocerus rhenanus* [5], those from Vallonnet, France [9] and Atapuerca TD6, Spain [10], ascribed to *Dama nestii vallonetensis*, shows in all the cases larger dimensions for Barranc de la Boella specimens. The comparison of the third lower molar with those from Barranco León (*Metacervocerus rhenanus*) shows a larger mesio-distal diameter (MDD) for La Boella, although the bucco-lingual diameter (BLD) is the same. We have also compared the average values for Dama vallonetensis and, as in the M1, this molar show a larger MDD. In consequence, and waiting for more resolutive material we have classified it as Cervidae indet. (middle size).

Comments: Unfortunately, because of the scanty and non resolutive anatomical material, we cannot determine this taxon.

Species: Cervidae indet. (large size)

Material: A radius fragment (BB09 C3 N1 O14 nº 5) and two pedicel fragments of large size (BB09 C3 N1 O14 nº 16 and BB09 EL FORN C3 NIV I O14 nº 8).

Three specimens from the level I, corresponding to large size species of deer, Cervidae indet. (large size), have been recovered.

Comments: As in the anterior case, and waiting for a more and better decisive fossils, we cannot classify this species.

**Order PERISSODACTYLA Owen, 1848**

Family RHINOCEROTIDAE Owen, 1845

Genus *Stephanorhinus* Kretzoi, 1942

Species *Stephanorhinus hundsheimensis* (Toula, 1902)

Comments: A mostly complete left hemimandible with P3-M3 (BB09 C3 NIV 3 UII), a left M3 (BB09 C3 N2 M11 nº 1) and a M2 (BB09 C3 N2 P14 nº 4).

Most of the Rhinocerotidae specimens from Barranc de la Boellawere found in the level 3, where it has been recorded a mostly complete left hemimandible with P3-M3, but the P4, M1 and M2 are broken in the lingual face. Another isolated M3 has been found in the same level, and one upper molar, M2, in the level 2.

Comments: The metric and morphologic comparison of the dental remains from Barranc de la Boella, with those of the Pleistocene *Stephanorhinus* species recorded in Europe (*S. etruscus*, *S. hundsheimensis*, *S. hemitoechus* and *S. kirchbergensis*) [11], shows that they fall in the variability of *Stephanorhinus hundsheismensis*, close to the specimens from Fuente Nueva 3 and Barranco León [12]. *S. hundsheimensis* is a typical species of the European late Villafrancian that survives until the base of the middle Pleistocene.

Family EQUIDAE Gray, 1821

Genus *Equus Linnaeus*, 1758

Species *Equus* sp.

Material: DP (BB09 C3 N2 M12 nº 7); left DP3-DP4 (BB09 C3 N1 N14 nº 3); left DP3-DP4 (BB08 C2 N2 P15 nº 8); left and right I1-I2-I3 (BB09 C3 N2 E10 nº 1) and a diaphysis fragment of radius (BB08 C2 N2 Q14 nº 15).

During the last four dig seasons, eleven teeth specimens ascribed to the Family Equidae have been recorded, five of them are deciduous and the other are six adult incisors. The postcranial record is only composed by one radius diaphyseal fragment.

Comments: It is not possible to classify the equid species recorded at Barranc de la Boella until more decisive anatomical material will be found.

**Order CARNIVORA Bowdith, 1821**

Family URSIDAE Gray, 1825

Genus *Ursus Linnaeus*, 1758

Species *Ursus* sp.

Material: 1 mostly complete proximal phalanx (BB08 C2 N2 R14 nº 7), together with several unrestored teeth that could not be analised.

Because of the scanty material with low anatomical resolution of this taxon, we have assigned this material to *Ursus* sp.

Comments: The taxonomy of early Pleistocene ursids has became complexed during last years, especially after the description of the species *Ursus dolinensis* at Gran Dolina of Atapuerca, Spain [13] and *Ursus rodei* at Untermassfeld, Germany [14], together with *Ursus etruscus*, well known in the Late Pliocene and early Early Pleistocene of Europe, including Venta Micena, Spain [15], and 'Ubeidiya, Israel [16,17]. Also, an archaic form of the speleoid form *Ursus deningeri* [9] was cited at Vallonnet (southeastern France), which is a typical species in most of the middle Pleistocene fossil assemblages of Europe.

Family CANIDAE Gray, 1821

Genus *Canis Linnaeus*, 1758

Species *Canis mosbachensis* Soergel, 1925

Material: Right M3 (BB08 C2 N2 P15 nº6).

It is a complete, slightly worn piece, corresponding to the species *Canis mosbachensis*.

Comments: *Canis mosbachensis*, a jackal-like dog, is a characteristic form of the European Late Villafranchian (Venta Micena, Untermassfeld, Vallonnet, Pirro Nord, Apollonia, ‘Ubeidiya and others, but also of the Galerian (see Martínez-Navarro et al., 2009, and references there in). It is more derived and of smaller size than the species of the basal Late Villafranchian Canis etruscus, which is recorded in different European sites, but especially in Italy, and also at Dmanisi [18,19]. *Canis mosbachensis* has been erroneously cited several times as *Canis arnensis*, which is another canid species of small-middle size recorded at the base of the Late Villafranchian, well known in Italy, with similar proportions and size to *C. mosbachensis*, but with different anatomy, especially in the molars, both upper and lower [17,20].

Family HYAENIDAE Gray, 1869.

Genus cf. *Pachycrocuta* Kretzoi, 1937

Species cf. *Pachycrocuta brevirostris* (Aymard, 1846)

Material: 28 coprolites of large size from unit II of la Mina.

Comments: The record of these coprolites documents the presence of a large size carnivore, a scavenger bone-cracker. Although we cannot classify it, most probably it has to be the large sized hyena *Pachycrocuta brevirostris*, well known in Europe during the Late Villafranchian, from 2.0 to 0.9-0.8 Ma.

*Paleoecological and Biochronological summary*

The faunal record of Barranc de la Boella represents one of the youngest fossil assemblages with Late Villafranchian fauna of Europe, including the proboscidean with generalistic feeding habits, grasses and leaves eater, *Mammuthus meridionalis*. The presence of this species shows that the site has a chronology earlier to the arrival into Europe of the inmigrant proboscidean coming from Africa*, Elephas (Palaeoloxodon) antiquus* [21], that will be present in the southern European biotopes during the middle Pleistocene and will survive until the base of the late Pleistocene. It is also recorded in Central Europe during the interglacial periods.

Although *E. (P.) antiquus* has been cited in the latest early Pleistocene of southern Iberian Peninsula, at Huéscar 1, dated 1.0-0.9 Ma [3], the adscription of this material to this species has been posteriorly questioned and it has been reclassified as *Mammuthus trogontherii* [22] and as *Mammuthus meridionalis* [23]. Because of this, the earliest clear record of *E. (P.) antiquus* in Europe is at the site of Slivia [24], in the early-middle Pleistocene transition, although most of the authors consider that the arrival of this species to Europe is around 0.6 Ma [21], at the chronology of Isernia La Pineta (Italia) [25].

The presence of *Hippopotamus antiquus* in this assemblage is also very significant. It is a species with total water dependence that shows derived anatomical characteristics, with elongated palate, and very raised nasal and orbital cavities, that show its great adaptation to aquatic environments, much more than the extant *species Hippopotamus amphibius*. Also, at the site of Venta Micena, southern Spain, it has been proved through a biogeochemical analysis that *H. antiquus* was only eating aquatic plants and not terrestrial ones [26]. *H. antiquus* is found in most of the Late Villafranchian and basal Galerian faunal assemblages (~1.6-0.6 Ma) of southern and central Europe.

The record of *Hippopotamus antiquus* informs of the presence of a huge quantity of water, within channels and pools, that also is very rich in vegetable resources, where the generalistic elephants such is *Mammuthus meridionalis*, can survive in good conditions, and where the presence of other ungulate species less water-dependent such rhinos (*Stephanorhinus hundsheimensis*), horses (*Equus* sp.), or deers, althoug they are present, they are not abundant.

These aquatic paleoenvironments, with abundant vegetation, are good places for the survival of carnivores like canids (*Canis mosbachensis*), ursids (*Ursus* sp.), or hyaenids (cf*. Pachycrocuta brevirostris*), thanks to the abundance of large ungulates. Of course, this same environment is good for hominins, with vegetable resources in forests of bank where to be protected, and animal resources by the presence of carcasses of megaherbivores and other ungulates. Similar environments, with presence of analogue species, can be observed at Buia, Eritrea (1.0 Ma) that are associated to a rich Acheulean lithic assemblage [8,27].

***References***

1. Martínez-Navarro B, Palmqvist P, Madurell J, Ros-Montoya S, Espigares MP, et al. (2010) La fauna de grandes mamíferos de Fuente Nueva-3 y Barranco León-5: estado de la cuestión. In: Toro I, Martínez-Navarro B, Agustí J, editors. Ocupaciones humanas en el Pleistoceno inferior y medio de la cuenca de Guadix-Baza. Sevilla: Junta de Andalucía. pp. 197-236.

2. Rook L, Martínez-Navarro B (2010) Villafranchian: a Plio-Pleistocene European large mammal biochronologic unit (its long story and remarks for its use). Quaternary International 219.

3. Mazo AV (1989) Nuevos restos de Proboscídea (Mammalia) en la cuenca de Guadix-Baza. In: Alberdi MT, Bonadonna FP, editors. Geología y Paleontología de la Cuenca de Guadix-Baza Trabajos sobre el Neógeno-Cuaternario. Madrid: Museo Nacional de Ciencias Naturales. CSIC. pp. 225-236.

4. Madurell-Malapeira J (2006) Estudi de les restes d'Hippopotamus antiquus (Hippopotamida, Mammalia) del jaciment del Pleistocè inferior de Cal Guardiola (Terrassa) [Master thesis]. Cerdanyola: Universitat Autònoma de Barcelona. 125 p.

5. Abbazzi L (2010) La fauna de cérvidos de Barranco León y Fuente Nueva-3. In: Toro I, Martínez-Navarro B, Agustí J, editors. Ocupaciones humanas en el Pleistoceno inferior y medio de la cuenca de Guadix-Baza. Sevilla: Junta de Andalucía. pp. 273-290.

6. Mazza P (1995) New evidence on the Pleistocene Hippopotamuses of Western Europe. Geologica Romana 31: 61-241.

7. Faure M (1986) Les Hippopotamidés du Pléistocène ancien d’Oubeidyeh (Israël). In: Tchernov E, editor. Les Mammifères du Pléistocène inférieur de la Vallée du Jordain a Oubeidiyeh. Paris: Mémoires et Travaux du Centre de Recherche Français de Jerusalem. pp. 107-142.

8. Martínez-Navarro B, Rook L, Segid A, Yosieph D, Ferretti MP, et al. (2004) The Large Fossil Mammals from Buia (Eritrea): Systematics, Biochronology and Paleoenvironments. Rivista Italiana di Paleontologia e Stratigrafia 110: 61-88.

9. Moullé P-E (1992) Les grands mammifères du Pléistocène inférieur de la grotte du Vallonnet (Roquebrune-Cap-Martin, Alpes Maritimes). Étude paleontologique des carnivores, equidés, suidés et bovidés. Paris: Museum National d’Histoire Naturelle de Paris.

10. van der Made J (1999) Ungulates from Atapuerca. Journal of Human Evolution 37: 389-413.

11. Lacombat F (2003) Etude des rhinocéros du Pléistocène de l’Europe méditerranéene et du Massif Central. Paléontologie, phylogénie et biostratigraphie. Paris: Muséum National d'Histoire Naturelle de Paris. 511 p.

12. Lacombat F (2010) Estudio paleontológico de Stephanorhinus hundsheimensis de Fuente Nueva-3 y Barranco León. In: Toro I, Martínez-Navarro B, Agustí J, editors. Ocupaciones humanas en el Pleistoceno inferior y medio de la cuenca de Guadix-Baza. Sevilla: Junta de Andalucía. pp. 237-246.

13. Garcia N, Arsuaga JL (2001) Ursus dolinensis: a new species of Early Pleistocene ursid from Trinchera Dolina, Atapuerca (Spain). Comptes Rendus de l'Académie des Sciences de Paris 332: 717-725.

14. Musil R (2001) Die Ursiden-Reste aus dem Unterpleistocezan von Untermassfeld. In: Kalhke RD, editor. Das Pleistozän von Untermassfeld bei Meiningen (Thüringen). Bonn: Rómisch-Germanisches Zentralmuseum. pp. 633-657.

15. Torres T (1992) Los restos de oso del yacimiento de Venta Micena (Orce, Granada) y el material de Ursus etruscus del Villafranquiense europeo. In: Gibert J, editor. Presencia humana en el Pleistoceno inferior de Granada y Murcia Proyecto Orce-Cueva Victoria (1988-1992). Orce: Ayuntamiento de Orce. pp. 87-106.

16. Ballesio R (1986) Les Carnivores du gisement Pléistocène d’Oubéidiyeh. In: Tchernov E, Guérin C, editors. Les Mammifères du Pleistocène inférieur de la vallée du Jourdain à Oubeidiyeh. Paris: Association Paléorient. pp. 63–92.

17. Martínez-Navarro B, Belmaker M, Bar-Yosef O (2009) The large carnivores from Ubeidiya (early Pleistocene, Israel): biochronological and biogeographical implications. Journal of Human Evolution 56: 514-524.

18. Sotnikova M, Rook L (2010) Dispersal of the Canini (Mammalia, Canidae: Caninae) across Eurasia during the Late Miocene to Early Pleistocene. Quaternary International 212: 86-97.

19. Vekua A (1995) Die Wirbeltierfauna des Villafranchian von Dmanisi und itere biostratigraphische Bedeutung. Jarhb Ger- Rónz Zentralmuseum Mainz 42: 77-180.

20. Martínez-Navarro B (2002) Presence of African large mammals (primates, carnivores and ungulates) in the Lower Pleistocene of the Middle East and Europe. Annales des Pays Hellèniques 39: 337-351.

21. Lister AM (2004) Ecological interactions of Elephantids in Pleistocene Eurasia: Palaeoloxodon and Mammuthus. In: Goren-Inbar N, Speth JD, editors. Human paleoecology in the Levantine Corridor. Oxford: Oxbow. pp. 53-60.

22. Davis P (2002) The stright-tusked elephant, Palaeoloxodon antiquus, in Pleistocene Europe. London: University College of London. 524 p.

23. Madurell-Malapeira J, Minwer-Barakat R, Alba DM, Garcés M, Gómez M, et al. (2010) The Vallparadís section (Terrassa, Iberian Peninsula) and the latest Villafranchian faunas of Europe. Quaternary Science Reviews 29: 3972-3982.

24. Palombo MR, Ferretti MP (2005) Elephant fossil record from Italy: knowledge, problems, and perspectives. Quaternary International 126–128: 107-136.

25. Coltorti M, Feraud G, Marzoli A, Peretto C, Ton-That T, et al. (2005) New 40Ar/39Ar, stratigraphic and palaeoclimatic data on the Isernia La Pineta Lower Palaeolithic site, Molise, Italy. Quaternary International 131: 11-22.

26. Palmqvist P, Gröcke DR, Arribas A, Fariña RA (2003) Paleoecological reconstruction of a lower Pleistocene large mammal community using biogeochemical (δ13C, δ15N, δ18O, Sr:Zn) and ecomorphological approaches. Paleobiology 29: 205-229.

27. Martíni F, Libsekal Y, Filippi O, Ghebre/Her A, Kashay H, et al. (2004) Characterization of lithic complexes from Buia (Dandiero basin, Danakil depression, Eritrea). Rivista Italiana di Paleontologia e Stratigrafia 110 (supplement): 99-132.

**Figure Legend**

**Figure S1.** *Mammuthus meridionalis* dental remains found at Barranc de la Boella in pit 1 locality level 2. Left: upper M1 (BB07 C1 N2 O12 nº 84). Rigth is upper M3 (BB07 C1 N2 P13 nº 115). Both scale bar 5 cm.
