# Supplementary material for: Age and Date for Early Arrival of the Acheulian in Europe (Barranc de la Boella, la Canonja, Spain)
Source: PLoS One. 2014 Jul 30;9(7):e103634. doi: 10.1371/journal.pone.0103634 (PMC4116235; doi:10.1371/journal.pone.0103634)
Supplement: Information S2 — Small mammals at Barranc de la Boella localities. Table S1, Average, maximum and minimum values for the length (L) and width (W) of the Mimomys savini m1 from early Pleistocene Iberian sites. (DOC) [file pone.0103634.s005.doc]

**Supporting Information S2. Small mammals at Barranc de la Boella localities.**

The Barranc de la Boella microvertebrate record comes from unit II of the EF (El Forn) and LM (La Mina) localities. The faunal list contains the taxa *Mimomys savini* and *Victoriamys chalinei*. The microvertebrate remains of *M. savini* consist of a first upper molar (M1), a first lower molar (m1) and a third upper molar (M3). The remains of *V. chalinei* consist of one m1 and one m2 recovered from LM.

**Systematic palaeontology**

**ORDER RODENTIA Bowdich, 1821**

FAMILY CRICETIDAE Rocheburne, 1883

Genus *Mimomys Frosyth* Major, 1902

Species *Mimomys savini* Hinton*, 1910*

The main diagnostic characteristics of the m1 of this species are as follows: a typical morphology of the tooth divided into a posterior lobe, five alternating triangles and an anterior lobe (triangles 4 and 5 (T4 and T5) with the anterior lobe formed by the anteroconid complex); the presence of roots (except in the earliest stages of life, before they have formed); enamel with *Mimomys*-type differentiation[1-4] cement in the re-entrant angles; T4 and T5 scarcely confluent; deep LRA3 and LRA4, producing a pronounced LSA4 and, depending on the age of the populations, a practically non-existent BRA3; and a tendency for the neck of the head of the anteroconid complex to be wide.

Material: *Mimomys savini* is represented at the Barranc de la Boella site by seven dental remains (two m1, three M3 and two M1) and one enamel fragment (Figure S2).

The first lower molars (one right m1 and one left m1) and the enamel fragment come from EF. These molars have little cement in the re-entrant angles, the zones on the occlusal plane without enamel are well defined and do not have enamel islets or the *Mimomys* ridge. The right m1 is slightly fractured in the anteroconid complex (missing part of the anterior lobe) and both roots are fractured, but formed.

Meanwhile, the M3 (two right M3s, one from LM and one from EF and one left M3 from EF) have occlusal plane zones without well-defined enamel and have thin cement in the re-entrant angles with the exception of the M3 from LM. The anterior lobes of the M3s from EF are broken.

Lastly, the M1s (one right and one left) documented in LM have thin cement in the re-entrant angle. The right M1 is broken and has a missing posterior triangle.

Comments: The absence of the enamel islet and *Mimomys* ridge in these few remains suggests a relatively modern M. *savini* biological population. The comparison of tooth width with other biological populations from other Iberian sites (Gran Dolina, Vallparadis, Fuente Nueva 3 and Barranco León) confirms this hypothesis. The evolution of this species leading to the increased size and width of the remains at Barranc de la Boella suggest that these populations have a chronology of less than 0.9 Ma, i.e. a chronology less than that of TD6 unit at Gran Dolina, Atapuerca, Spain [5-7]. This observation has not been possible in the comparisons of m1 lengths (L) with other sites because the values obtained from the remains at Barranc de la Boella correspond to a population older than 0.9 Ma. Due to the reporting bias caused by the small sample sizes of *M. savini* at Barranc de la Boella, comparison with other sites is not viable for the purpose of estimating chronology. (Figure S3 and Table S1).

Genus *Victoriamys* Martin, 2012

Species *Victoriamys chalinei* Alcalde, Agustí y Villalta, 1981

The main diagnostic characteristics of the m1 of this species are as follows: a typical morphology of the tooth divided into a posterior lobe, five alternating triangles and an anterior lobe; triangles 4 and 5 are largely confluent with one other and with the anterior lobe, which are very wide and short and have an extensive enamel-free zone.

Material: one right m1 and one right m2 from LM. The m1 element is broken on the anterior lobe and triangles T4 and T5 are totally confluent. Both remains have well-defined enamel-free zones on the occlusal plain and abundant cement in the re-entrant angles. This cement is more abundant at the base of the tooth than at the crown. The thick enamel is undifferentiated between the anterior and posterior parts of the triangles

Comments: It has not been possible to adopt a comparative approach with other samples as only one m1 skeletal remain has been recovered.

**Paleoecological and Biochronological summary**

*Mimomys savini* was an ancestor of *Arvicola mosbachensis* [8,9] the first representative of the genus of today’s aquatic rodents (*A. terrestris* and *A. sapidus*). *M. savini* went extinct around 0.5 Ma ago, opening up an ecological niche that was filled by the genus *Arvicola*. This replacement suggests that both genera lived in the same environment and in the same habitat. Therefore, the presence of *M. savini* suggests the existence of areas with slow and quiet continental water streams, on muddy or sandy fluvial channel bars and abundant herbaceous or shrub vegetation.

*Victoriamys chalinei* is found only on the Iberian and Italian peninsulas close to continental Mediterranean-sea margins and was cited as *Allophaiomys chalinei* [10]. The geographic distribution of *V. chalinei* suggests an association with the Mediterranean climate. Therefore, the presence of both species (*M. savini* and *V. Chalinei*) suggests a landscape with abundant water and herbaceous vegetation.

From a biochronological point of view, the coincidence of these species located at unit II of the Barranc de la Boella site within the Iberian Peninsula biozone of *V. Chalinei* described by Cuenca-Bescos et al.gives us a chronological range of between 0.9 Ma ago and 0.78 Ma ago [7].

**References**

1. Heinrich W-D (1978) Zur biomestrischen Erfassung eines Evolutiontrends bei Arvicola (Rodentia, Mammalia) aus dem Pleistozän Thüringens. Säugetierkundliche Informationen 2: 3-21.

2. Heinrich WD (1987) Neue Ergebnisse zur Evolution und Biostratigraphie von Arvicola (Rodentia, Mammalia) im Quartär Europas. Zeitschrift für geologische Wissenschaften 15: 389–406.

3. Koenigswald WV, Kolfschoten TV (1996) The Mimomys-Arvicola boundary and the enamel thickness quotient (SDQ) of Arvicola as stratigraphic markers in the Middle Pleistocene. In: Turner C, editor. The early Middle Pleistocene in Europe. Rotterdam: Balkema. pp. 211–226.

4. Koenigswald WV, Sander MP, Leite MB, Mörs T, Santel W (1994) Functional symmetries in the schmelzmuster and morphology of rootless rodent molars. Zoological Journal of the Linnean Society 110: 141-179.

5. Falguères C, Bahain J-J, Yokoyama Y, Arsuaga JL, Bermudez de Castro JM, et al. (1999) Earliest humans in Europe: the age of TD6 Gran Dolina, Atapuerca, Spain. Journal of Human Evolution 37: 343-352.

6. Berger GW, Pérez-González A, Carbonell E, Arsuaga JL, Bermúdez de Castro JM, et al. (2008) Luminescence chronology of cave sediments at the Atapuerca paleoanthropological site, Spain. Journal of Human Evolution 55: 300-311.

7. Cuenca-Bescós G, Rofes J, López-García JM, Blain H-A, De Marfá RJ, et al. (2010) Biochronology of Spanish Quaternary small vertebrate faunas. Quaternary International 212: 109-119.

8. Chaline J, Brunet-Lecomte P, Montuire S, Viriot L, F. C (1999) Anatomy of the arvicoline radiation (Rodentia): palaeogeographical, palaecoecological history and evolutionary data. Annales zoologici fennici 36: 239-267.

9. Chaline J, Sevilla P (1990) Phyletic gradualism and developmental heterochronies in a European Plio-Pleistocene Mimomys linage (Arvicolidae, Rodentia). In: Fejfar O, Heinrich WD, editors. Evolution, Phylogeny and Biostratigraphy of Arvicolids. Rohanov: Geological Survey. pp. 85-98.

1. Laplana C (1999) Presencia de Microtus (Allophaiomys) chalinei Alcalde, Agust|. y Villalta, 1981 (Arvicolidae, Rodentia) en el yacimiento de Bagur-2 (Pleistoceno inferior, Girona, España). Treballs del Museu Geologic de Barcelona 8: 25-32.

Supporting Information Figure Legends

**Figure S2.** A: M3, M1 and broken M1 from *M. savini* found from unit II in pit 2 or la Mina locality. B: Bucal, occlusal and labial views of m1 (top) and m2 (bottom) from *V. chalinei* sampled in unit II at pit 2 or la Mina site. C, Bucal, occlusal and labial views of m1 from *M. savini* (top two) and enamel remains of one m1 and two M3’s from M. *savini* (bottom, left to right) recorded in pit 3 or el Forn unit II. Scale bar 1 mm.

**Figure S3.** Graphic representation comparing the length (L) and width (W) of the Barranc de la Boella sample and other documented *M. savini* specimens from selected Iberian sites. These Iberian sites are arranged from older (left) to younger (right) (except Barranc de la Boella).

Supporting Information Table

**Table S1.** Average, maximum and minimum values for the length (L) and width (W) of the *Mimomys savini* m1 from early Pleistocene Iberian sites.

| **Measurements** | O | GD4B | GD5b | GD5a | GD6-3 | GD6-2 | GD6-1 | VP | BB |
| --- | --- | --- | --- | --- | --- | --- | --- | --- | --- |
| **L mean** | 3.23 | 3.34 | 3.46 | 3.48 | 3.43 | 3.58 | 3.61 | 3.48 | 3.31 |
| **L max** | 3.4 | 3.6 | 3.78 | 3.82 | 3.96 | 3.8 | 3.82 | 3.81 | 3.34 |
| **L min** | 2.92 | 3.25 | 3.04 | 3.11 | 2.87 | 3.36 | 3.26 | 3.14 | 3.29 |
| **W mean** | 1.41 | 1.44 | 1.46 | 1.49 | 1.49 | 1.55 | 1.51 | 1.53 | 1.55 |
| **W max** | 1.6 | 1.52 | 1.67 | 1.67 | 1.63 | 1.69 | 1.64 | 1.69 | 1.63 |
| **W min** | 1.26 | 1.31 | 1.27 | 1.26 | 1.23 | 1.41 | 1.38 | 1.46 | 1.48 |
| **N** | 132 | 10 | 137 | 103 | 102 | 4 | 9 | 22 | 2 |

^a^ Keys: O, Orce basin sites (Barranco León and Fuente Nueva 3); GD, Gran Dolina (Sierra de Atapuerca)-units; VP: Vallparadís (Tarrassa); BB: Barranc de la Boella unit II (la Canonja).
